# Chi hotspots trigger a conformational change in the helicase-like domain of AddAB to activate homologous recombination

**DOI:** 10.1093/nar/gkv1543

**Published:** 2016-01-13

**Authors:** Neville S. Gilhooly, Carolina Carrasco, Benjamin Gollnick, Martin Wilkinson, Dale B. Wigley, Fernando Moreno-Herrero, Mark S. Dillingham

**Affiliations:** 1 School of Biochemistry, University of Bristol, Bristol BS8 1TD, UK; 2 Department of Macromolecular Structures, Centro Nacional de Biotecnología, Consejo Superior de Investigaciones Cientificas, 28049 Cantoblanco, Madrid, Spain; 3 Institute of Cancer Research, Chester Beatty Laboratories, 237 Fulham Road, London SW3 6JB, UK; 4 Section of Structural Biology, Department of Medicine, Imperial College London, South Kensington Campus, London SW7 2AZ, UK

## Abstract

In bacteria, the repair of double-stranded DNA breaks is modulated by Chi sequences. These are recognised by helicase-nuclease complexes that process DNA ends for homologous recombination. Chi activates recombination by changing the biochemical properties of the helicase-nuclease, transforming it from a destructive exonuclease into a recombination-promoting repair enzyme. This transition is thought to be controlled by the Chi-dependent opening of a molecular latch, which enables part of the DNA substrate to evade degradation beyond Chi. Here, we show that disruption of the latch improves Chi recognition efficiency and stabilizes the interaction of AddAB with Chi, even in mutants that are impaired for Chi binding. Chi recognition elicits a structural change in AddAB that maps to a region of AddB which resembles a helicase domain, and which harbours both the Chi recognition locus and the latch. Mutation of the latch potentiates the change and moderately reduces the duration of a translocation pause at Chi. However, this mutant displays properties of Chi-modified AddAB even in the complete absence of *bona fide* hotspot sequences. The results are used to develop a model for AddAB regulation in which allosteric communication between Chi binding and latch opening ensures quality control during recombination hotspot recognition.

## INTRODUCTION

The repair of double-stranded DNA breaks by homologous recombination first requires resection of the DNA end to generate a long 3′-ssDNA overhang suitable for RecA/Rad51-dependent strand exchange. During DNA end resection in bacterial cells, the recognition of recombination hotspot sequences by helicase-nuclease enzymes is a key mechanism for the production of this recombinogenic DNA molecule (1–5). For example, the *B. subtilis* AddAB helicase-nuclease complex catalyzes rapid and processive DNA end resection using a single Superfamily 1A (SF1A) helicase motor and two nuclease domains which are dedicated to the cleavage of each of the nascent unwound strands of DNA (2,6). Resection is limited by Chi sequences (5′-AGCGG in *B. subtilis*) which promote recombination by modulating the nuclease activity. A current model is that binding of Chi within the enzyme complex sequesters the 3′-terminated strand and prevents it from engaging with the AddA nuclease domain, thereby resulting in nuclease activity attenuation beyond Chi (7,8). Continued unwinding and degradation of the 5′-terminated strand by the AddB nuclease domain produces a long 3′-ssDNA overhang; the substrate required for RecA-mediated strand exchange (9). This resection reaction is analogous to that performed by the well-studied RecBCD enzyme, although there are also significant differences between the two systems (see (2,10) for reviews).

**Figure 1.**
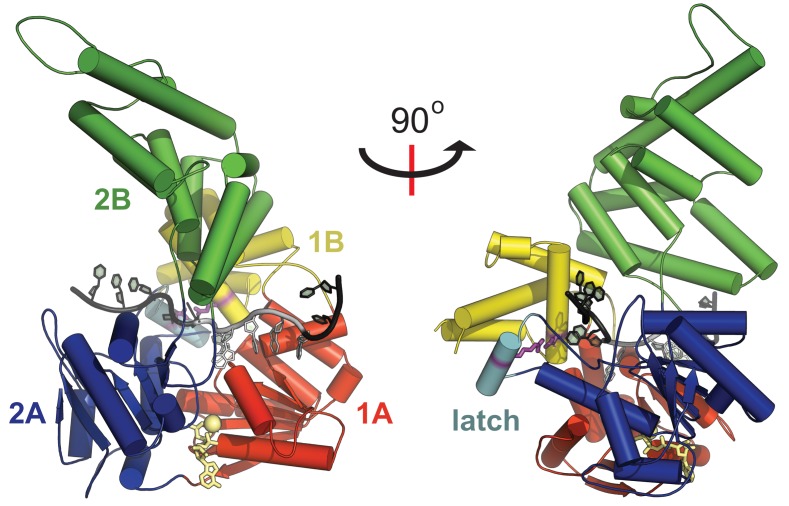
An ionic latch is a unique and highly conserved feature of the helicase-like domains found in DNA break resection complexes. The N-terminal domain of the AddB subunit (PDB: 4CEJ) shares structural homology with the UvrD-like class of Superfamily 1 DNA helicases. The four subdomain structure contains a tandem repeat of two RecA folds (1A and 2A) which act here as a scanner for Chi sequences but which, in *bona fide* helicases, act as an ATP-dependent DNA motor. The ssDNA is shown in gray with the bound Chi sequence 5′-AGCGG in a lighter shade, with an AMP-PNP molecule and a Mg^2+^ ion shown in pale yellow. In addition to their unusual and specialized function as a Chi scanner, the core RecA folds include a unique addition in the form of a loop–helix–loop insertion in domain 2A (cyan helix). This structure, which we refer to as the ionic latch, is held in position by many ionic interactions including an absolutely conserved salt bridge between E129 and R629 (purple, stick format).

Upon arriving at Chi sequences, both AddAB and RecBCD complexes pause and then decrease translocation rate beyond Chi (11–14). These phenomena are somewhat mechanistically distinct, reflecting differences in the domain composition and organisation of the two complexes (for further details see (2,3,10)). However, the proposed mechanism for binding of Chi sequences is similar. In both cases, biochemical and structural analysis suggests that inactivated helicase domains found in the N-terminal regions of AddB and RecC recognise the Chi sequence (7,15–17). These ‘helicase-like’ domains share the same overall topology as canonical Superfamily I (SF1) helicases, but lack most or all of the characteristic motifs required for classical DNA translocation and unwinding activity (Figure 1). Instead, the helicase fold appears to have been co-opted for use as a ‘scanner’ for specific ssDNA sequences. Evidence in support of this view is provided by the observation that the location of the Chi binding site in AddB and RecC is precisely equivalent to the ssDNA tracking site of a conventional SF1 helicase (7,16,17). The structures of these inactivated helicase domains are unique in that they contain an additional short loop–helix–loop insertion in domain 2A (Figure 1, cyan helix). We refer to this structure as an ‘ionic latch', because it forms intramolecular contacts to the 1B domain that are stabilized by salt bridges, including one formed between the absolutely conserved residues E129 in domain 1B and R629 in the latch (Figure 1, purple residues). Intriguingly, the latch structure occludes a narrow channel formed between the Chi recognition domain of AddB and the motor domain of AddA (Supplementary Figure S1), and an analogous channel is found in RecBCD (7,17). A ‘channel-bypass' model for events that follow Chi recognition (in both AddAB and RecBCD) predicts the ungating of this channel to allow the formation of a growing ssDNA loop beyond Chi that is protected from degradation (7,10,17,18). In support of this idea, Chi remains tightly bound within the AddAB complex following recognition (8), Chi-dependent DNA loops have been inferred (12,19) and mutations which destabilize the latch structure enhance the efficiency of Chi recognition (7,17). Recent work provided evidence for a Chi-dependent conformational change in the RecC subunit of ReBCD, but this was not interpreted in terms of the channel bypass model (20). However, there is no direct evidence for the necessary conformational change in an AddAB-type enzyme, or for its control through the latch structure.

In this work, we have studied the properties of AddAB complexes containing mutations that disrupt either (or both) the Chi-binding site and the ionic latch. These mutations decrease and increase the Chi recognition efficiency of the complex respectively. We show that disruption of the ionic latch improves Chi recognition even in mutants that cannot bind Chi efficiently, results in the formation of hyper-stable AddAB-Chi complexes, and potentiates a Chi-dependent conformational change. The conformational change deprotects the inactivated helicase domain of AddB from proteolysis, and is consistent with a repositioning of the ionic latch to open a new exit channel. We propose a model for Chi recognition in which the latch acts as a quality control mechanism during DNA break repair by creating an energy barrier that is used to discriminate between Chi and other non-specific sequences.

## MATERIALS AND METHODS

### Proteins and DNA substrates

Wild type and mutant AddAB enzymes were expressed and purified as described previously (7). Tailed DNA substrates were prepared by 5′-labeling of linearized pADG6406–1; before removing excess nucleotide, Exonuclease III was added to a final concentration of 0.55 units μl^−1^ and incubated at 20°C for 2.5 min. The tailing reaction was stopped by the addition of EDTA to a final concentration of 20 mM before Phenol:Chloroform:Isoamyl Alcohol extraction and isopropanol precipitation. Pellets were resuspended in 10 mM Tris·Cl pH 8.0 and then passed through a S400 column to remove residual nucleotides. Tailed DNA was then cleaved with the restriction endonuclease SpeI (NEB) following the manufacturer's instructions. This yields a single binding site for AddAB ensuring unidirectional processing of the DNA substrate. Experiments performed in this way reduce complications associated with competing reactions on the same substrate and/or collisions between two AddAB complexes.

### DNA break resection and exonuclease chase assay

DNA substrates (1.6 nM) were incubated with 2 μM SSB tetramer and 1 mM ATP at 37°C for 2 min in a buffer containing 20 mM tris-acetate (pH 7.5) 2 mM magnesium acetate and 1 mM DTT. Reactions were initiated by the addition of excess AddAB (4 nM). Reactions were quenched after 4 min by transferring 10 μl of the reaction to an equal volume of 2× Stop Buffer (Proteinase K 1 mg/ml, 100 mM EDTA, 10% (w/v) Ficoll 400, 5% (w/v) SDS, 0.125% (w/v) bromophenol blue, 0.125% (w/v) xylene cyanol). Enzyme concentrations are shown in respective figure legends. All assay products were run on 1% (w/v) agarose gels, dried and reaction products analyzed as described previously (7).

Exonuclease chase assays are modified versions of the break resection assay, and were based on the method of Chedin *et al*. (8). Briefly, when an excess of Exonuclease I (ExoI) is added following DNA break resection assays (performed as above), the rate of cleavage of the Chi-fragment by ExoI is limited by the dissociation of AddAB from Chi. Therefore, the disappearance of the Chi-fragment due to the addition of ExoI indirectly measures the rate of AddAB dissociation from Chi.

### Limited proteolysis

Limited proteolysis was conducted during DNA processing by AddAB. In these reactions AddAB was pre-incubated with the DNA substrates 5-Chi or 5-ChiScramble in standard reaction buffer at 37°C for 2 min. 5-Chi and 5-ChiScramble were made by annealing the oligonucleotides 5-Chi tail with 5Chi_2 and 5-ChiScramble with 5-ChiScramble_2 (MWG) in water (for sequences see Supplementary Information). This was accomplished by heating to 95°C and cooling to 20°C at a rate of 0.23°C/h in a thermocycler. This yielded two dsDNA substrates that possess a 30nt ssDNA tail in order to prevent binding of AddAB to one DNA end.

The processing of these DNA substrates was initiated by the addition of ATP to a concentration of 1 mM. After 20 s, α-Chymotrypsin was added to a concentration of 1 μg/ml. 10 μl aliquots were removed at indicated time points and added to 10 μl of stop buffer (62.5 mM Tris–Cl pH 6.8, 2% (w/v) SDS 25% glycerol, and 0.01% (w/v) bromophenol blue, 1 mM PMSF) and immediately placed at 95°C for 2 min prior to electrophoresis through 4–15% SDS-PAGE gels (Bio-rad). Nuclease attenuation assays confirmed that these substrates are resected by AddAB in a manner regulated by Chi as would be expected (Supplementary Figure S3).

### Magnetic tweezers translocation assay

Single molecule translocation experiments were conducted at ambient temperature (typically ∼22°C) as described previously (14). In summary, all measurements were carried out using a tailor-made magnetic tweezers microscope in a flow cell with a channel height of 200 μm, at a previously calibrated force of 3 pN and a camera sampling rate of 60 Hz. Biotinylated wild type or mutant AddAB proteins were pre-bound to streptavidin-coated 1 μm beads (MyOne Dynabeads, Invitrogen) and dsDNA substrates containing a well-defined region of ten consecutive Chi sequences at ∼4.6 kb from the near DNA end (see substrate Chi10-Rev-For in (14)). Protein–DNA constructs were subsequently tethered to the flow cell surface via single antibody–antigen interactions. Translocation activity of AddAB was triggered by flushing reaction buffer with 1 mM ATP at a volumetric rate of ∼1 μl/s. The corresponding position-versus-time traces were inferred from optically detected height changes of magnetically trapped beads with respect to reference beads nonspecifically attached to the glass surface. To reduce Brownian noise, raw data were filtered to 3 Hz for representation and for analysis of pause frequency/duration and translocation rate.

Experiments at 37°C were accomplished using a temperature controller that has been described previously (21) and otherwise following the procedure illustrated above. Briefly, to raise the temperature in the vicinity of AddAB, a thermal control assembly consisting of two customized heating circuits – one for the objective and one for the sample stage—was implemented in the microscope. To yield a stable value of 37ºC with ∼0.5ºC of accuracy inside the flow cell, heater setpoints around 39ºC and 41ºC, respectively, had to be calibrated first. After an initial warm-up period, the system was given at least 30 min of stabilisation time prior to tethering of AddAB–DNA beads. To minimize temperature fluctuations during the measurements, ATP-containing buffer was preheated to 37°C before injection. To minimize non-specific attachments of proteins and beads we pre-incubated the fluid cell with 0.1 mg/ml BSA. Data analysis was performed as stated above, but taking into account the temperature dependence of the rise-per-bp value at the applied force, needed to convert absolute distances into positions along the DNA track (14,21).

### Triplex displacement translocation assay

Triplex displacement experiments were performed and analyzed as described previously (13). Briefly, DNA substrates containing a single triplex binding site were annealed overnight at 20°C to a 4-fold excess of 5′-TAMRA labeled triplex forming oligonucleotide (TFO) (see supporting information for TFO sequence) in a buffer containing MES (12.5 mM, pH 5.5) and MgCl_2_ (10 mM). Free TFO was removed via S-400 (GE Healthcare) spin column chromatography. DNA substrates (2 nM) were incubated with streptavidin (100 nM) in a buffer containing BSA (100 μg/ml), tris acetate (25 mM, pH 7.5), magnesium acetate (2 mM) and DTT (1 mM). AddAB enzymes (10 nM) were incubated in this solution at 37°C for 2 min before mixing against a solution containing AddA^K36A^B (200 nM), ATP (1 mM) BSA (100 μg/ml), Tris-acetate (25 mM, pH 7.5), magnesium acetate (2 mM) and DTT (1 mM). These two solutions were rapidly mixed and the resulting fluorescence was recorded at 37°C in a stopped-flow instrument with a xenon-mercury lamp (TgK Scientific). TAMRA was excited at 547 with 5.4 nm slit widths and the fluorescence above 570 nm was recorded. Data were normalized to the fluorescent endpoint by dividing each data point by the total signal change. Data obtained on Chi-free DNA was analyzed semi-quantitatively by fitting to the sum of two offset exponentials. This yields the reported values for the first phase triplex release amplitude (see (13) for details). Data obtained on Chi-containing DNA was not analyzed in this manner due to the underlying complexity of the translocation process for the latch mutant complex. Instead, the first phase amplitude was crudely estimated from the absolute fluorescence amplitude at *t* = 2 s.

## RESULTS

### Chi binding is allosterically coupled to an ionic latch

**Figure 2.**
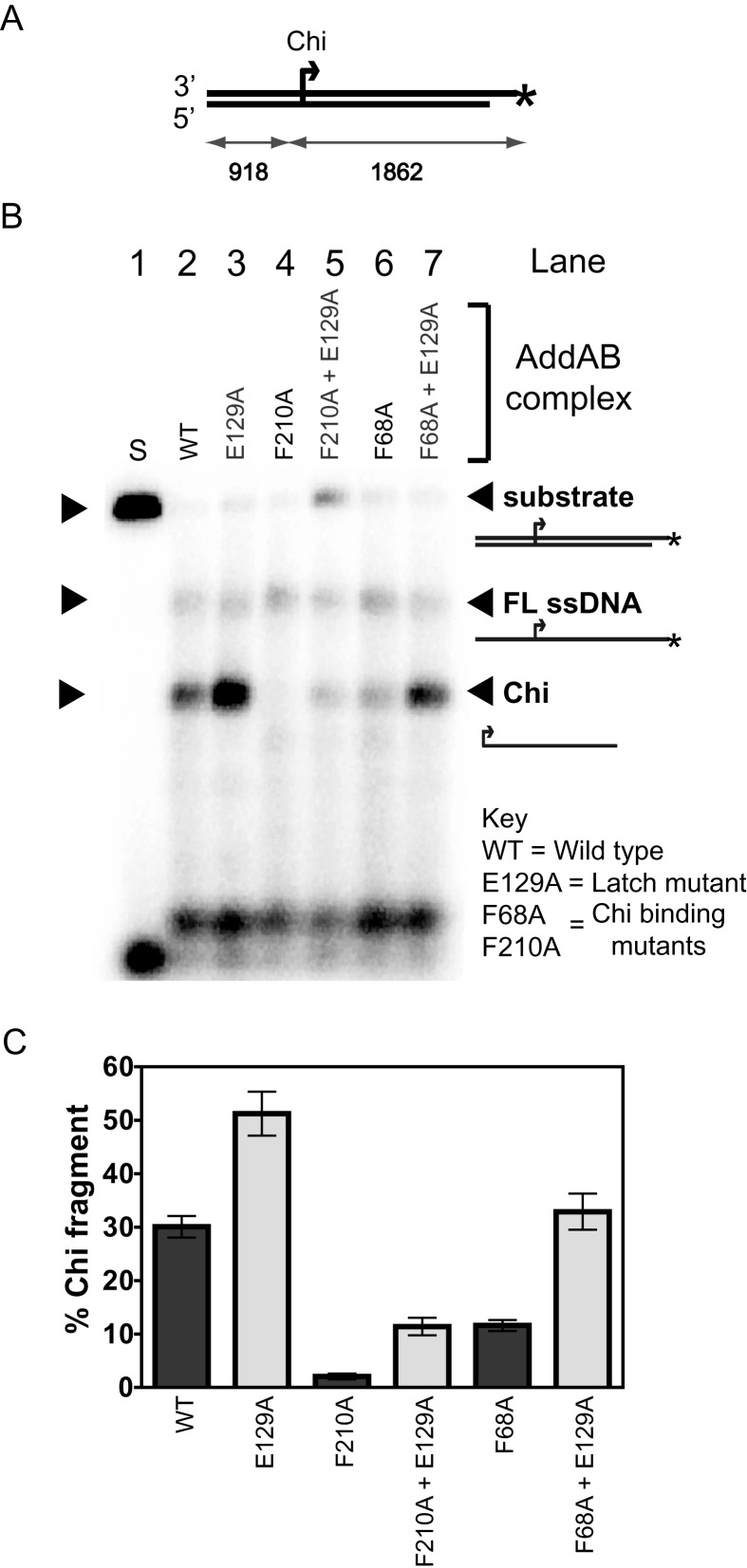
Disruption of the ionic latch can rescue a Chi binding deficiency. (**A**) Schematic of the DNA substrate used in these experiments. AddAB can only enter from the left of these substrates as shown. The position of a single Chi sequence is highlighted. The asterisk marks the position of a radiolabel. (**B**) DNA end resection assays in which 1.6 nM tailed DNA molecules were processed by excess (4 nM) AddAB enzymes in the presence of 2 μM SSB and 1 mM ATP at 37°C for 30 s. Gels have been uniformly contrast enhanced to aid visualization of reaction products. The mutations present in each enzyme are indicated above the lanes and on the key. (**C**) Quantification of total Chi fragment. Error bars represent the standard error of the mean (SEM) derived from three independent experiments.

To investigate the potential role of the latch structure in mediating Chi recognition, we investigated the apparent Chi recognition efficiency and AddAB–Chi complex stability using two ‘Chi-binding mutants' (F68A and F210A in AddB) and a ‘latch mutant' (E129A in AddB). These decrease and increase, respectively, the Chi recognition efficiency as measured by nuclease attenuation assays (7). It should be noted that the E129A mutation is located in AddB domain 1B, rather than in the latch itself, and is designed to destabilize the closed conformation of the latch observed in all crystal structures of AddAB so far (Figure 1). The idea that Chi binding is energetically coupled to latch disruption predicts that mutants with a low affinity for Chi might be partially or fully rescued by the latch mutation. To test this hypothesis, we first compared Chi fragment production by the Chi binding mutants (7), with their double mutant counterparts (AddAB^F210A+E129A^ and AddAB^F68A+E129A^) that also included the latch mutation (Figure 2). The resection of DNA breaks by wild type and mutant complexes was investigated with DNA substrates that either did, or did not, contain Chi sequences in defined positions. All of the mutant proteins resected Chi-free DNA in the same manner as wild-type AddAB ((7); Supplementary Figure S2). On Chi-containing DNA substrates (Figure 2A), the mutation of the latch increases the yield of Chi fragment in an otherwise wild type background by ∼2-fold (Figure 2B, compare lanes 2 and 3). As observed previously (7), the AddAB^F210A^ mutant is severely defective in its response to Chi (∼15-fold reduced, lane 4), whereas the AddAB^F68A^ mutant exhibits a moderate defect (∼2.5-fold, lane 6). In line with our predictions, both double mutant complexes show significant increases in Chi fragment yield relative to the single mutants. Indeed, the amount of Chi fragment produced by the AddAB^F68A+E129A^ complex mutant is rescued to wild type levels (Figure 2C).

**Figure 3.**
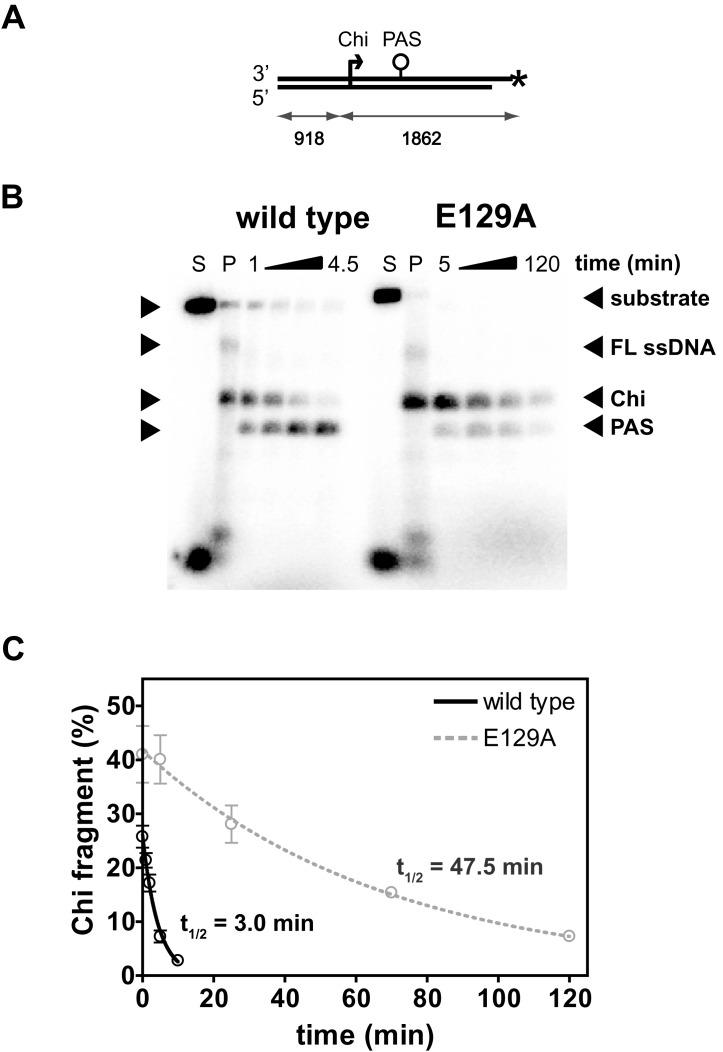
Disruption of the ionic latch results in the formation of hyper-stable AddAB-Chi complexes. (**A**) Schematic of the DNA substrate used in these experiments. AddAB can only enter from the left of these substrates as shown. The positions of a single Chi sequence and primosome assembly site (PAS) are highlighted. The asterisk marks the position of a radiolabel. (**B**) Exonuclease chase assay to measure stability of the AddAB:Chi complex. Lane S shows unprocessed substrate DNA, and lane P shows the products of AddAB resection assays, which is used to estimate the initial yield of Chi fragment. Subsequent time points (indicated) are from the Exonuclease chase reaction start time. 1.6 nM DNA molecules were processed by 4 nM AddAB enzymes in the presence of 2 μM SSB and 1 mM ATP at 37°C. ExoI was added to a final concentration of 0.8 units/μl. Black triangles mark the DNA substrate, full length (FL) ssDNA, the Chi fragment and the primosome assembly site (PAS; a region of secondary stucture that is ExoI resistant (8)). Gels have been uniformly contrast enhanced to aid visualisation of reaction products. (**C**) Quantification of total Chi fragment as a function of time. Both data sets were fit to a single exponential decay to zero. The half life (*t*_$\frac{1}{2}$_) is indicated. Error bars represent the SEM derived from three independent experiments.

We next compared the dissociation rate of wild type and mutant AddAB complexes from Chi (Figure 3). The off-rate of AddAB from Chi can be determined by ‘chasing' a DNA break resection reaction with a large excess of Exonuclease I (ExoI) (8). AddAB that remains specifically bound to Chi following recognition protects the 3′-end of the ssDNA fragment, such that the Exo I degradation rate is limited by the dissociation rate of AddAB from Chi. This effectively provides a time-resolved footprint of AddAB at Chi. Note, that the ssDNA is not fully degraded because a primosome assembly site (PAS) downstream of the Chi sequence forms stable secondary structure that inhibits Exo I-mediated ssDNA degradation (Figure 3A). Before addition of the exonuclease chase, wild-type AddAB resects the radiolabeled dsDNA substrate to produce the Chi fragment amongst a smear of single-stranded DNA resection products (Figure 3B, lane P). Following addition of the chase exonuclease, the smear of resection products is rapidly degraded but the Chi-fragment is long-lived. (8)). The kinetics of Chi fragment depletion are well-fit by a single exponential decay (Figure 3C) and this yields a half-life for the wild type AddAB:Chi complex of ∼3 min which is similar to a previously published value (6). The behavior of the latch mutant is dramatically different in two respects. Firstly, consistent with the data presented above in Figure 2, the latch mutant shows an increased initial response to Chi (7), producing about twice the initial yield of Chi fragment compared to wild type (Figure 3C, at *t* = 0). Secondly, and most strikingly, the complex formed between the latch mutant and Chi is dramatically stabilized (by ∼15-fold; *t*_1/2_ ∼45 min). Note that the Exo I remains fully active during the extended time course required to measure latch mutant dissociation from Chi (data not shown). Mutation of the Chi binding locus has the opposite effect; the complexes formed between AddAB^F68A^ and Chi dissociate more quickly than wild type (Supplementary Figure S2, *t*_1/2_ ∼0.8 min). However, further mutation of the latch produces a similar overall stabilizing effect (∼15-fold) as it does in a wild type context, with AddAB^F68A+E129A^ also forming hyper-stable AddAB-Chi complexes (*t*_1/2_ ∼13 min). These results provide direct evidence that the *wild type* latch structure antagonizes Chi recognition, both in terms of AddAB:Chi stability and the observed efficiency of recognition. Therefore, we hypothesized that destabilisation of the latch structure is allosterically coupled to Chi binding, and that the response to Chi involves a trade-off between the binding energy available in the AddAB-Chi interaction and an energy penalty associated with movement of the ionic latch. We next sought to detect such a Chi-dependent conformational change directly, and to test whether any such change was modulated by the structural integrity of the ionic latch.

### Chi recognition induces a conformational change in the helicase-like domain of AddB that is potentiated by disruption of the latch

In order to determine whether the recognition of Chi elicits a conformation change in AddAB, limited proteolysis experiments were conducted following DSB resection reactions on substrates with and without Chi sequences. Small oligonucleotide-based molecules were used to facilitate reactions being performed at the high concentrations of protein required for these experiments. We initially confirmed that these novel substrate DNAs elicited the normal response to Chi sequences using nuclease attenuation assays (Supplementary Discussion 1 and Supplementary Figure S3). Then, using chymotrypsin, we performed limited proteolysis on AddAB enzymes after they had been allowed to interact with either Chi-free or Chi-containing DNA substrates in the presence of ATP.

**Figure 4.**
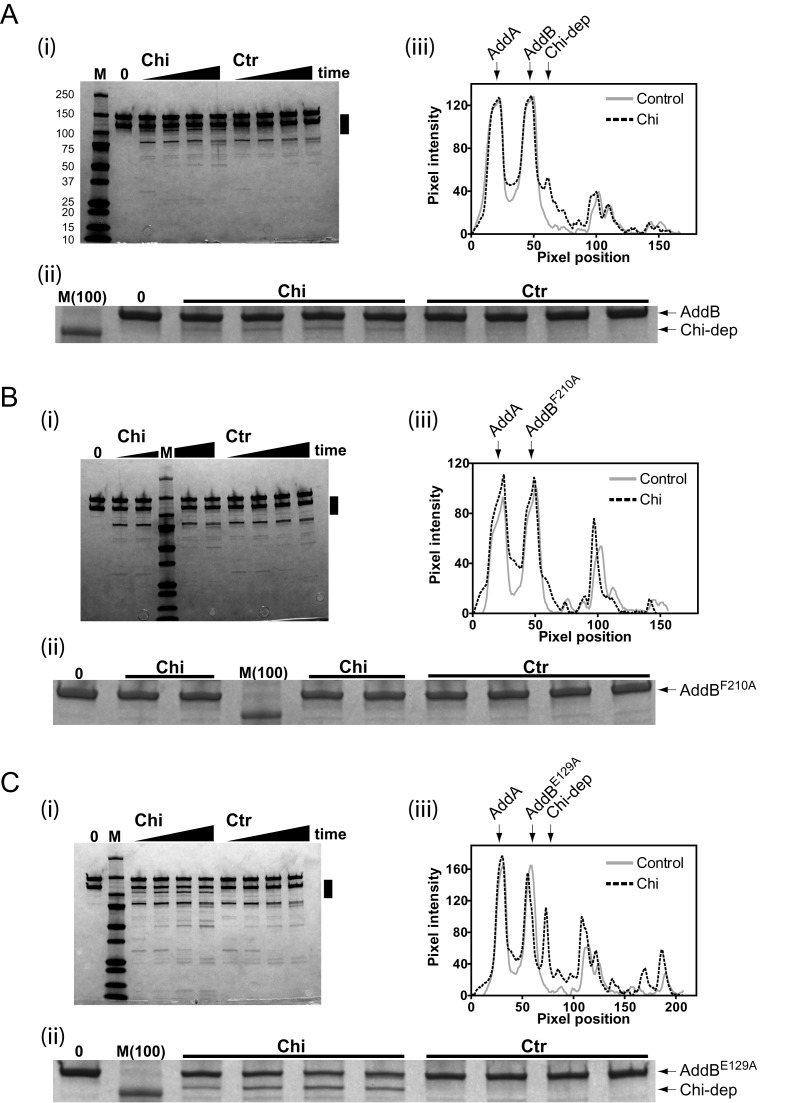
A Chi-dependent conformational change is potentiated by mutation of the ionic latch. (**A**) Limited proteolysis experiments with wild type AddAB. (i) Wild-type AddAB enzymes (1 μM) were prebound to DNA substrates (4 μM) that either did (Chi) or did not (Ctr) contain Chi sequences at 37°C for 2 min. Resection reactions were initiated by the addition of ATP (1 mM). After 30 s, α-Chymotrypsin was added to a final concentration of 1 μg/ml. Reaction time points are 1, 2, 4 and 10 min. The zero timepoint shows the position of the uncut AddA and AddB polypeptides. Reactions were terminated by the addition of loading buffer and subsequent heating at 95°C for 2 min. Samples were then analyzed by SDS-PAGE. Lane M denotes protein markers (size indicated on left of gel). (ii) Zoomed-in version of the gel shown in the panel above highlighting the region marked by a black bar. The positions of full length AddB and the Chi-dependent fragment are highlighted. (iii) The lane profiles for the 10 min timepoints are shown to the right, extending from ∼150 to ∼75 kDa. The lane profiles are similar for the Chi and control DNA (compare solid and dotted lines) except for a Chi-dependent band at ∼110 kDa (indicated). (**B**) The same experiment was conducted with AddAB^F210A^, a mutant complex that is severely defective in binding Chi (7). (**C**) The same experiment was conducted with the latch mutant complex (AddAB^E129A^). Note an increased yield of the Chi-dependent product in this case.

The wild-type enzyme is proteolyzed into a complex series of discrete bands most of which are identical regardless of whether Chi-containing or Chi-free DNA substrates are employed (Figure 4). However, at least one faint band of ∼110 kDa appears uniquely in experiments with DNA that contains correctly-oriented Chi sequences, and never in control experiments in which the Chi sequence is ‘scrambled' (Figure 4A, marked with an arrow in panel ii). Although it is a very minor product of the proteolysis reaction, this band is reproducible (e.g. see also Supplementary Figures S4 and S5) and could therefore be a digestion product originating specifically from Chi-modified AddAB enzyme, which we shall refer to below as AddAB*. Note that, when using these short oligonucleotide fragments as substrates, the yield of Chi fragments as a percentage of total input DNA is low (∼17%) meaning that the putative conformational change we are seeking to detect would only have occurred in a minority of the AddAB present (Supplementary Figure S3). Formation of the Chi-dependent proteolysis product was dependent upon ATP-dependent DNA translocation and the added protease, and was not associated with loss of an Fe–S cluster that is known to be important for structural integrity of the complex (Supplementary Figure S4 (22)). Importantly, in further support of our assertion, the band of interest does not appear when the Chi binding mutant AddAB^F210A^ is proteolyzed, regardless of the presence or absence of Chi sequences in the substrate DNA (Figure 4B). This confirms that the band of interest is dependent upon Chi recognition, as opposed to some unexpected peculiarity of the Chi-containing substrates. Critically, when the same experiment was performed with the latch mutant complex, not only was the band of interest recovered for the Chi-containing substrates, but its yield was approximately doubled relative to wild type (from ∼6% of total input to ∼11%; Figure 4C). This shows that disruption of the ionic latch potentiates a Chi-dependent conformational change, and is consistent with the enhanced Chi recognition and stability associated with the latch mutant that we have observed above.

**Figure 5.**
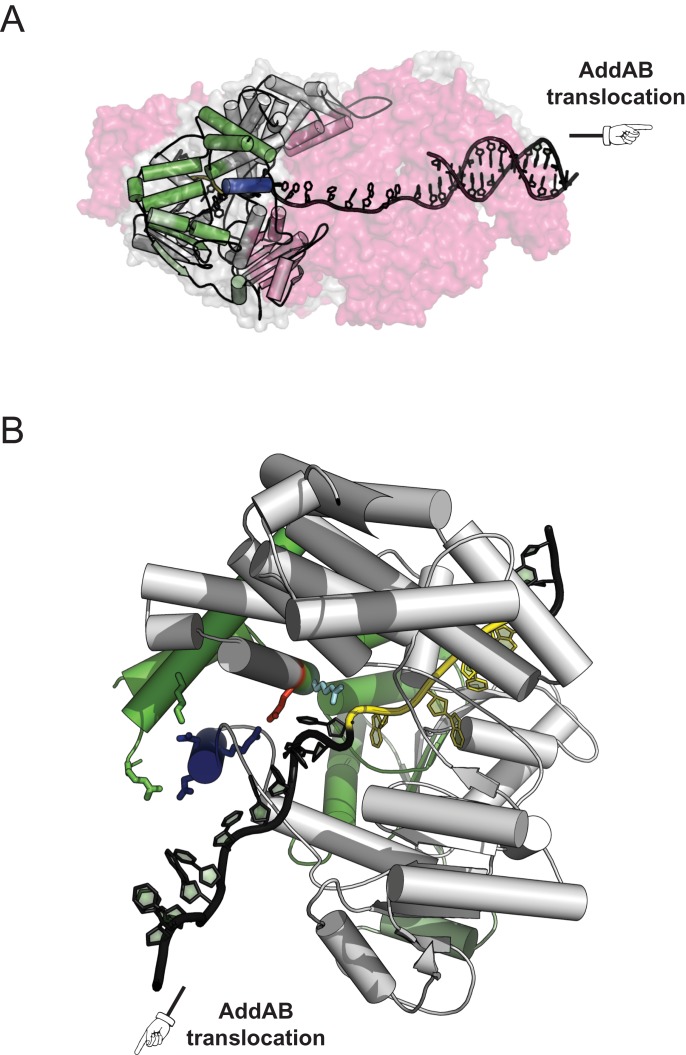
The Chi-dependent cleavage site maps close to the latch binding pocket in AddB. (**A**) The AddAB complex is viewed from below, with translocation of the enzyme along the DNA (black) from left to the right (see also Supplementary Figure S1 for orientation). The surface is transparent and colored red (AddA) and light gray (AddB). The N-terminal helicase-like domain of AddB is also shown in gray cartoon format, with the latch helix in blue. We deduce that the Chi-dependent cleavage of AddB occurs in the green colored region which encompasses the entire latch binding pocket. (**B**) Alternative orientation showing only AddB and DNA, and highlighting intramolecular interactions of the latch helix, and a potential pathway for allosteric communication with the Chi binding locus. The enzyme complex would move along the black DNA towards the bottom left of the page, but stable binding of the Chi sequence (yellow) in AddB prevents this. Movement of the latch would open an alternative exit, allowing a loop of black DNA to leave the complex while the yellow Chi sequence remains bound, and make the latch binding pocket region (green) susceptible to proteolytic cleavage post-Chi as observed. The latch is held in place by ionic interactions between D627, D628, R629 (all blue) and R153 (green), K161 (green), E129 (red) respectively. Note also that the arginine residue neighbouring E129 (R132, cyan) interacts directly with the phosphate group immediately to the 5′-side of the Chi sequence.

To map the position of the Chi-dependent proteolysis product, the band of interest was excised and subjected to *in situ* tryptic digestion followed by peptide identification by mass spectrometry. This approach was used because the protein was not amenable to N-terminal sequencing even after optimisation of the product yield (Supplementary Discussion 2 and Supplementary Figure S5). The results were compared with a control, in which an equivalently positioned gel fragment was excised from an experiment performed with Chi-free DNA (Supplementary Figure S5a). Many peptides were detected from the C-terminal region of AddB, starting from residue 265 (latch mutant experiment) or residue 309 (wild type experiment) and providing good coverage of the rest of the polypeptide to near the C-terminus (Supplementary Figures S5b and S5c). In comparison, either very few or no peptides were detected from the AddA polypeptide, and the control experiment detected very few peptides from either AddA or AddB (Supplementary Figure S5b). Given the molecular weight of the band of interest (110 kDa +/− 10 kDa) we conservatively estimate that the Chi-specific cleavage product arises from a single cleavage site between residues 130 and 297, in good agreement with the earliest detected peptides. The cleavage site must therefore be within domain 1A of the core inactivated helicase region of AddB or the proximal side of domain 1B (see Figure 1 for domain organisation). This region is close to residues that directly interact with Chi (7,23) and includes the part of AddB that is contacted by the latch in the pre-Chi conformation (Figure 5, green region). Therefore, the mapping results are consistent with the idea that Chi recognition causes a conformational change in which the latch moves from its current position, exposing a nearby site in AddB for cleavage by chymotrypsin. This simple mechanism would explain why disruption of the pre-Chi conformation of the latch increases Chi recognition and improves detection of the conformational change. However, we cannot exclude the possibility that a different conformational change occurs in the same region of AddB and/or that additional conformational changes occur elsewhere in the complex upon Chi recognition, as may be required to load RecA (24,25).

We have recently presented a crystal structure of an AddAB-Chi complex in which the latch remains in the closed conformation (23). We argued that this structure represents an initial ‘Chi encounter complex’, which is responsible for the pause at Chi observed in single molecule experiments, and that continued ATP hydrolysis might be required to observe subsequent conformational changes to the full AddAB* state (see also the Discussion). To test this idea, we performed limited proteolysis on AddAB-DNA complexes equivalent to those used for crystallography, but allowing the ATP hydrolysis that is not possible *in crystallo* (Supplementary Figure S4c). These experiments showed the production of the same Chi-dependent proteolysis product, supporting the suggestion that the crystallographic snapshots are indeed physiologically relevant and are *en route* to the AddAB* state. As observed above, this protease sensitivity was strongly potentiated by the latch mutation (E129A) and also by the mutation R629A in the latch itself, which replaces the arginine that pairs with E129 to stabilize the closed latch conformation (Figure 1).

### Disruption of the latch moderately shortens the measured pause time at Chi sequences

**Figure 6.**
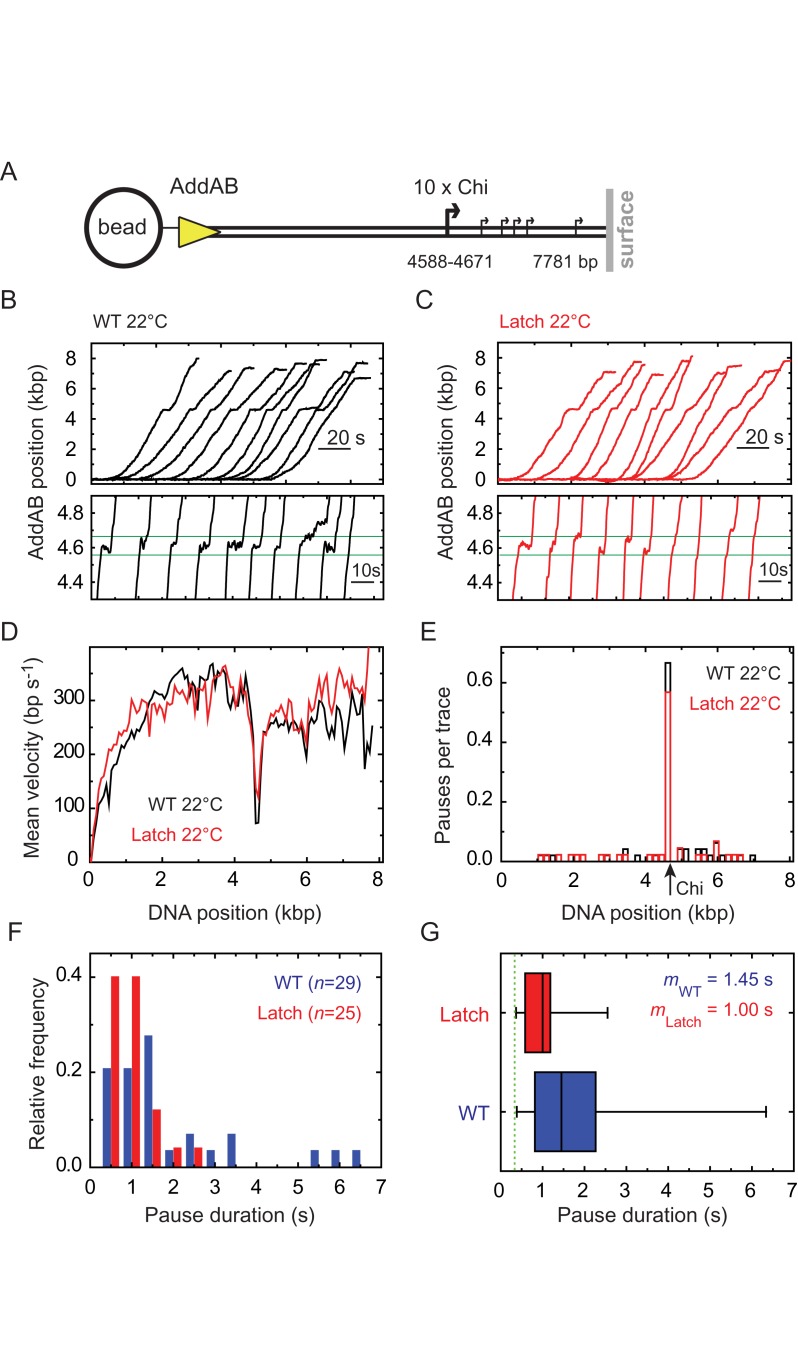
Disruption of the ionic latch moderately reduces the pause duration at Chi. (**A**) Single molecule DNA translocation experiments were performed using a DNA substrate containing a Chi locus consisting of an array of 10 closely spaced Chi sequences at ∼4.6 kbp from the free DNA end (14). Ten representative traces smoothed to 3 Hz for (**B**) wild-type AddAB and (**C**) AddAB^E129A^ are shown offset on the same graph. Both experiments were conducted at temperatures around 22°C. A zoomed-in region around the position of the Chi locus (marked with two green lines) is shown as lower panels in (B) and (C). (**D**) Mean velocity as a function of position on DNA for wild-type and latch mutant AddAB measured at room temperature. Each curve corresponds to the average of at least 40 individual velocity traces. The sharp dip in translocation rate is due to Chi sequence recognition. (**E**) Pauses per translocation trace distribution. Typically, 6 out of 10 traces present a clear pause at the Chi locus for both wild type and latch AddAB mutant. (**F**) Histogram of the pause duration at Chi for wild-type AddAB (blue) and latch mutant AddAB^E129A^ (red) at ∼22°C. The data are binned in 0.5 s windows and represented as thinner bars with 0.2 s offsets for clarity. The total number of events *n* is stated for both cases. (**G**) Box and whiskers plot of the data shown in (F). The box boundaries represent the 25^th^ and 75th percentile, respectively, and the whiskers span the whole pause duration range. The black line inside each box indicates the median *m*, whose numerical values are significantly different according to a Mood's median test (*P* = 0.014). The green dotted line represents the temporal resolution of the magnetic tweezers instrument (14) below which pauses cannot be detected.

Using a single molecule magnetic tweezers approach, we previously showed that the wild-type AddAB complex undergoes a non-exponentially distributed pause (∼1.8 s at ∼20°C), when it translocates through a locus containing 10 Chi sequences (14). This was interpreted as reflecting multiple kinetic steps that are required to restart translocation following Chi recognition. This work also showed that, following recognition, translocation beyond Chi was slower. In this study, to determine whether the opening of the latch structure contributes to the total pause time at Chi, we performed equivalent single molecule experiments with the latch mutant complex (Figure 6).

The substrate used for translocation was a DNA fragment of ∼7.7 kb, containing ten closely-spaced Chi sequences starting at 4588 bp from the AddAB entry point (Figure 6A). A total number of 48 and 44 single molecule translocation traces were obtained at 22°C for AddAB and AddAB^E129A^ respectively, and representative examples are shown in Figure 6B and C. The wild type and latch mutant translocated with high processivity, at the same average rate (∼300 bp s^−1^) before Chi, and at a slightly decreased rate after Chi as expected (Figure 6D). Moreover, both showed a similar pause frequency at Chi (66% and 57%, respectively) (Figure 6E). Given that the mutant complex is actually significantly better at *responding* to Chi sequences (see above), this result indicates that the latch mutation does not affect the initial recognition of Chi sequences that arrests DNA translocation, but must instead promote a subsequent step leading to formation of a ‘mature' Chi recognition complex with enhanced stability. In comparison, the frequency of pausing outside of the Chi locus was very low, and was also similar for both complexes (Figure 6E). The pause duration histograms are not normally distributed, at least in part because the measurements are limited by the temporal resolution of the instrument (Figure 6F). A box and whiskers plot shows that the measured pauses are shorter in the latch mutant compared to the wild type (median/mean pause durations of 1.00/0.97 and 1.45/1.92 s, respectively, Figure 6G). Given the non-normality of the data and the presence of outliers, a nonparametric Mood's median test was used to investigate the equivalence of the two data sets. This suggests that there is a significant difference between the median pause duration of wild type and mutant AddAB (*P* = 0.014).

Single molecule experiments with the wild type and latch mutant were repeated at 37°C, where the pause duration is markedly decreased and pauses are detected less frequently because they are often shorter than the time resolution of the instrument (13) (Supplementary Figure S6). In addition to the expected increase in mean translocation rate for both enzymes (∼1000 bp s^−1^; Supplementary Figure S6d), a difference between them was observed at the Chi locus. Some pauses at Chi are observed for wild type AddAB (4 of 38 traces) whereas the latch mutant showed no detectable pauses at all (Supplementary Figure S6e). This reduced pause frequency is also clearly reflected by the lack of a sharp drop in mean velocity at the Chi locus for AddAB^E129A^ (Supplementary Figure S6d, compare black and red traces). Taking into account the data collected at a lower temperature, it is likely that the latch mutant complex does still pause at Chi, but that the duration is now always below the detection limit of the instrument (<0.33 s). In summary, our data suggest that the kinetics of latch opening contribute to the time spent at Chi before resumption of translocation, but other steps clearly remain (at least for the measurements at 22°C) that account for the majority of the dwell time at recombination hotspots.

### Disruption of the latch allows promiscuous conversion to a Chi-modified state

Based upon the experiments presented above, the biochemical properties of the latch mutant complex might be considered paradoxical, in the sense that they are *better* than wild type. We hypothesized that the latch mutant might display a defect in some aspect of its regulation by Chi. For example, it might illegitimately behave akin to the Chi-modified form, as has been shown for a similar mutation in RecBCD (17).

In gel-based Chi recognition assays, we found no evidence for the formation of novel Chi-like fragments when using Chi-free DNA substrates (e.g. Figures 2 and 3). However, such events would potentially occur at very common or even random sequences on the DNA and with low frequency, and would be extremely difficult to detect with conventional assays. Additionally, MT experiments did not show increased pausing away from the Chi-locus that might be attributed to promiscuous Chi recognition (Figure 6E), but this could simply reflect the limited time resolution of the apparatus. Therefore, to very sensitively detect such events, we exploited a known difference in the translocation rates of AddAB and AddAB* (13,14). AddAB enzymes that have recognised Chi move more slowly along DNA and this gives rise to biphasic triplex displacement kinetics in stopped-flow translocation assays (13). In these assays, a fluorescently-labeled triplex forming oligonucleotide is displaced by the translocating AddAB enzyme. The observed lag in the displacement kinetics indirectly provides information on the rate of movement along DNA of the whole enzyme population. On substrates that are free of Chi sequences, the wild type complex displaces triplexes in a single exponential phase that is offset on the time axis by the duration of translocation, a value that is inversely proportional to translocation rate (13). When Chi sequences are positioned in between the free DNA end and the triplex, there is a dose-dependent decrease in the amplitude of the first phase of triplex displacement, and a compensatory increase in a second (delayed) phase of triplex displacement (see (13)). This second phase of triplex displacement is caused by a slower population of AddAB (i.e. AddAB*) that has recognised the Chi sequence. Therefore, the presence of a delayed phase of triplex displacement is indicative of conversion to the AddAB* form. Note that the total delay time includes a contribution from the pause at Chi, but is dominated by the effect of slower translocation beyond Chi (discussed in (13)), such that it is not possible to measure small changes to the pause time in this assay.

**Figure 7.**
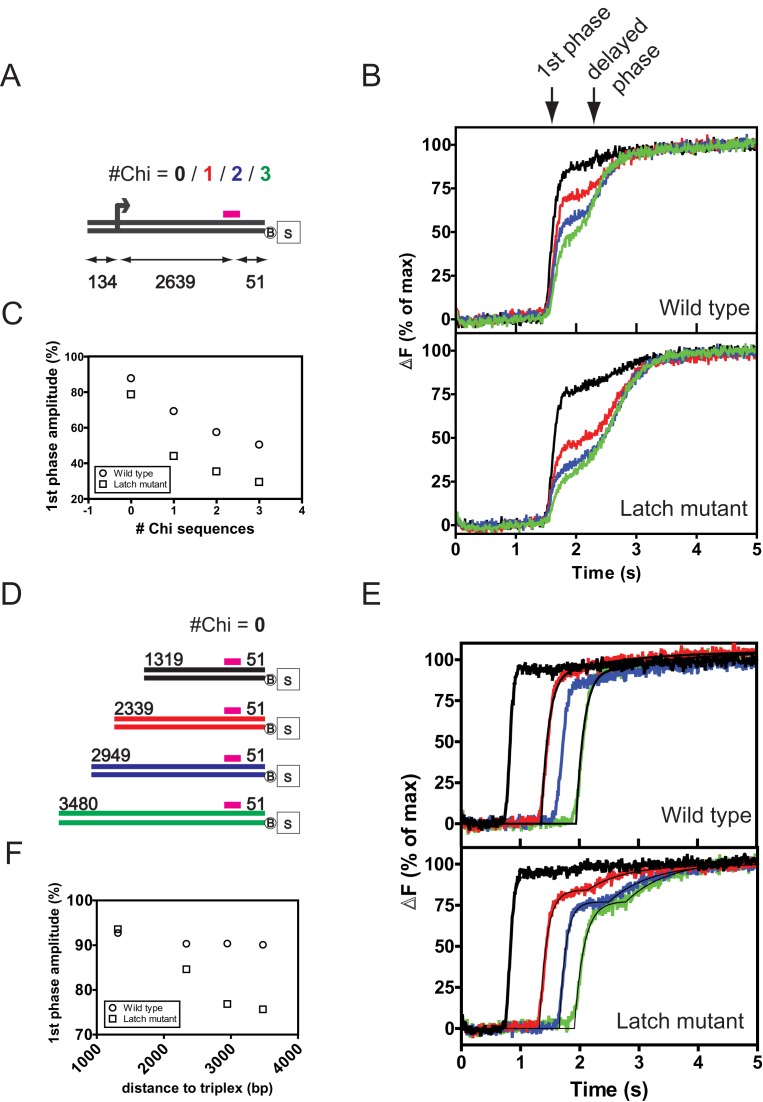
Disruption of the ionic latch leads to dysfunctional regulation of DNA translocation. (**A**) Schematic of substrates used in triplex displacement experiments to test the effect of Chi recognition on DNA translocation. The positions (in base pairs) of a Chi locus containing between zero and three Chi sequences, and of the triplex (magenta line) are indicated. The distal end of the substrate is blocked by a biotin-streptavidin complex to ensure unidirectional translocation. (**B**) Triplex displacement curves on the substrates shown in (A) using either wild type or mutant AddAB complexes. The curves are biphasic, with the first phase of triplex displacement representing enzymes that have not recognised Chi, and the second ‘delayed phase’ representing the enzyme population that has slowed down following recognition of Chi (13). (**C**) The first phase amplitude plotted against the number of Chi sequences for wild type and latch mutant AddAB complexes. (**D**) Chi-free DNA substrates with different distances between the free DNA end and the triplex. (**E**) Triplex displacement curves on the substrates shown in (D) using either wild type or latch mutant AddAB complex as indicated. (**F**) Data were fit as in (13) to extract amplitudes for the first phase of triplex displacement (see text for discussion). In all experiments above, 1 nM DNA molecules were prebound by 4 nM (saturating) AddAB enzymes. Reactions were initiated by mixing with an equal volume of ATP (1 mM) and AddA^K36A^B (100 nM) at 37°C. The mutant complex acts as a trap to ensure that only a single round of translocation can be observed in the experiment (13). Data is the average of at least 3 transients and normalized to the endpoint of fluorescence. The wild type data shown in this figure are reproduced from reference (13) with permission.

Triplex displacement traces produced by the latch mutant are markedly different to those of wild-type complex (Figure 7). When comparing substrates with different numbers of Chi sequences (Figure 7A), we see that the latch mutant responds to Chi in the same way as wild type (by slowing down translocation) but does so more efficiently (Figure 7B and C). For example, on a substrate with three Chi sequences, ∼50% of wild-type AddAB is converted to the slow moving AddAB* form, whereas ∼70% of the latch mutant complex is delayed in reaching the triplex (Figure 7C). This observation would be expected based on the improved Chi recognition associated with the latch mutation. However, we also observed that, even on Chi-free DNA, a larger proportion of the latch mutant complex translocates slowly, similar to AddAB* (Figure 7B and C, compare black traces in Figure 7B). To investigate this behavior and its mechanistic basis more closely, we performed additional experiments with Chi-free DNA in which the triplex was placed at greater distances from the AddAB entry point (Figure 7B). For wild type AddAB, the triplex is released largely in a single exponential phase for all of the substrates (Figure 7E and F). In contrast, for the latch mutant the relative amplitude of the first phase of triplex displacement clearly decreases with the distance between the DNA end and the triplex (Figure 7F), suggesting that a conversion to AddAB* occurs during translocation along Chi-free DNA. The absolute amplitudes of triplex displacement for wild type and latch mutant complexes are similar, suggesting that both enzymes display high processivity like wild type and consistent with our single molecule data (Supplementary Figures S7e and S7f). The behavior we have observed is consistent with a range of models whereby, at one extreme, the latch mutant stochastically switches into the AddAB* form or, at the other, it recognises degenerate Chi-like DNA sequences as if they were Chi. In either scenario, the longer the DNA substrate, the more of the AddAB^E129A^ population is able to isomerize into AddAB* before reaching the triplex. However, we observed very little effect of destabilizing Chi binding in the latch mutant complexes (see Supplementary Figure S7 for data and discussion). Therefore, it is most likely that mutation of the latch allows a stochastic isomerisation of the latch mutant into the Chi-modified form of the enzyme during translocation.

In summary, although the latch mutant is able to recognise Chi sequences more effectively than wild-type enzyme, the ensuing responses (which include slower translocation) appear to have become mis-regulated. These translocation data are independently corroborated by real-time DNA unwinding assays, which show that the latch mutant complex is also better at unwinding DNA than the wild type, a property that it shares with Chi-modified AddAB (19) (Supplementary Discussion 3 and Supplementary Figure S8). Together, the results show that the latch mutant enzyme shares biochemical properties associated with the Chi-modified form of the complex, even in the complete absence of *bona fide* Chi sequences.

## DISCUSSION

Following recombination hotspot recognition, the AddAB helicase–nuclease can unwind several kilobases of DNA beyond the Chi sequence, while remaining stably bound to Chi itself, thereby protecting DNA downstream of the hotspot from further cleavage and promoting recombination (8,19,26). The current structural model accommodates this observation by proposing the formation of a ssDNA loop downstream of Chi that must escape the enzyme complex via an alternative exit channel. The crystal structure of AddAB identified a possible channel between the AddA motor and the Chi recognition domain of AddB (Supplementary Figure S1; (7)). Occluding this putative alternate exit is a loop-helix-loop structure that protrudes from the 2A domain of AddB (Figure 1, cyan helix). The helix has been hypothesized to act as a molecular latch that maintains the alternative exit channel in a closed conformation before Chi is recognised (7,17). In support of this idea, mutation of an absolutely conserved amino acid that holds the latch in a closed confirmation (AddAB^E129A^) results in an *increased* response to Chi in nuclease attenuation assays ((7) and this work). This mutation can partially or fully rescue the Chi-recognition proficiency of AddAB with mutations in the Chi binding locus that reduce the affinity for the specific hotspot sequence. Moreover, time-resolved footprinting experiments revealed that complexes formed between the latch mutant complex and Chi are dramatically tighter than their wild type counterparts. Together, these results support the idea that Chi binding is allosterically coupled to a conformational change at the latch. Experiments investigating the role of an analogous latch structure in the RecBCD helicase-nuclease also concluded that it is allosterically coupled to the binding of Chi sequences (17). Moreover, destabilisation of the latch resulted in the recognition of variants of the canonical 8 base Chi sequence. However, in contrast to the case with AddAB, there was no apparent increase in the recognition of *bona fide* Chi sequences.

Direct evidence for a Chi-dependent conformational change is provided by limited proteolysis experiments. These reveal a unique digestion product in enzymes that have recognised Chi sequences. The site that becomes protease sensitive was mapped to part of the inactivated helicase domain of AddB, including the Chi binding locus itself and a region of domain 1B to which the latch helix is engaged in the closed confirmation (see Figure 5). Therefore, a simple possibility to explain our data is that the latch moves to a new position or becomes disordered following Chi recognition to promote the opening of an alternative exit channel for the ssDNA loop. However, we emphasize that any conformational change or deprotection of the surface of the AddB inactivated helicase domain is consistent with our data. Recent experiments with the RecBCD complex have also provided evidence for a conformation change using a similar proteolysis strategy (20). Using a proxy complex for the Chi-modified form of the enzyme, the authors detected a conformational change resulting in protease sensitive sites within the region 270–313 in RecC. Given the significant structural similarity of AddB and RecC, this result is broadly consistent with the one obtained herein. However, on the basis of additional SAXS and crosslinking experiments, this protease sensitivity was interpreted as reflecting a deprotection of RecC that resulted from the movement of the RecB nuclease domain. We currently have no evidence for a large conformational change in the nuclease domain of AddA (∼RecB), and all currently available crystal structures show the nuclease domain of AddA residing in a position on a different surface of AddB, where it is suitably positioned to cleave the 3’-terminated DNA strand. However, it should be noted that AddAB and RecBCD display different overall architectures, especially with respect to the organisation of nuclease domains (2).

The conformational transition we observe is thought to be reflected in the pausing of AddAB (and RecBCD) that is observed at Chi sequences (see (10,27) for reviews), because the complex cannot move forward from Chi until an alternative exit is open. Consistent with this idea, if the latch is destabilized, the complex displays a reduced pause time at Chi. However, a significant pause remains in the latch mutant, suggesting either that our mutation does not fully ‘unlock' the latch and/or that other rate limiting steps (perhaps associated with extrusion of the DNA loop) might be required to exit the paused state. An additional complexity in interpreting these data is that we have measured the pause at a Chi locus containing ten hotspot sequences, and it may represent failed as well as successful Chi recognition attempts (14). It is therefore also possible that there are less failed Chi recognition attempts for the latch mutant, and this could give rise to the smaller pause duration.

**Figure 8.**
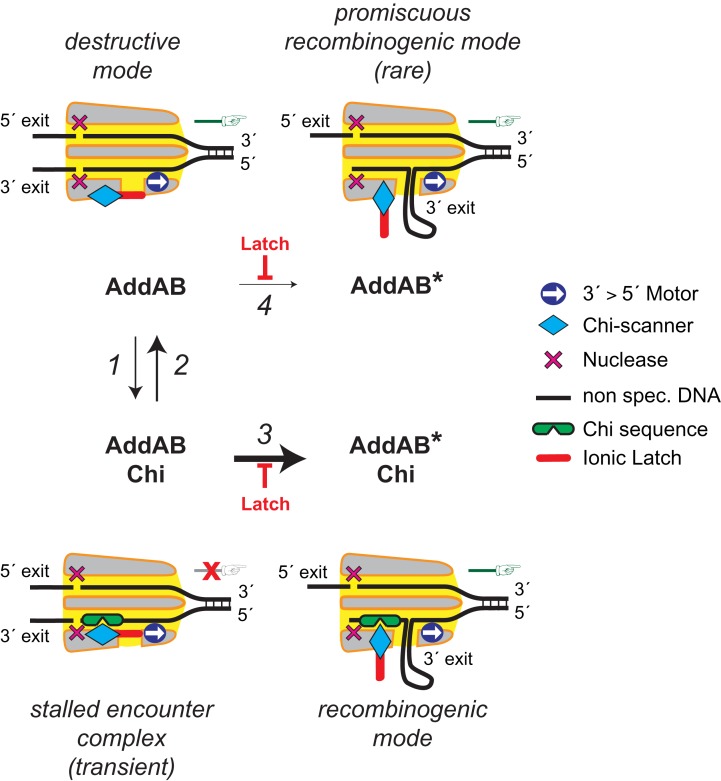
A two-state ‘gated-scanner model' for recombination hotspot recognition. A cut-through cartoon representation of the AddAB complex (gray surface, with yellow internal channels) is shown translocating from left to right (green hand) with DNA in black. Different functional modules (3′→5′ helicase motor, Chi scanner and nuclease domains) are indicated within the channels for the two DNA strands (see key). Prior to recognition of Chi sequences, the enzyme acts in a *destructive mode* (top left). The latch is in the closed position (‘AddAB state’) and the two DNA strands are guided past their respective nuclease domains and then out of the complex via primary exit channels. During translocation, Chi (or Chi-like) sequences occasionally arrive within the scanner and are transiently bound while the latch remains in the closed state (*step 1*), forming the *stalled encounter complex* (bottom left). If the pentameric DNA sequence is not optimal then this state is unstable, so the DNA is released and the enzyme quickly returns to the destructive mode with the latch still remaining in the closed state (*step 2*, indicated with thicker arrow). If the DNA is a *bona fide* Chi sequence (5′-AGCGG) then this state is stable, and binding is allosterically coupled to movement of the latch into the open position ‘AddAB* state’ (*step 3*, this is conceptually illustrated by the diamond-shaped scanner fitting into the notch in the Chi sequence). This forms the *recombinogenic mode* (bottom right) in which translocation can restart while Chi remains bound, because an alternative exit for the 3′-terminated DNA loop opens between the motor and the Chi scanner. In this ‘gated-scanner model’, opening of the latch in the absence of Chi sequences to form the promiscuous recombinogenic mode is possible (*step 4*), but energetically unfavourable (indicated with thin arrow). However, mutations that destabilize the latch (e.g. AddB E129A) would lower the energetic barrier to the formation of AddAB*, resulting in better recognition of Chi sequences at the expense of mis-recognition events. Mutations in the Chi binding site (e.g. AddB F210A) decrease the response to Chi by increasing the unbinding rate of Chi from the scanner (*step 2*). The model shown here was developed on the basis of this and previous work (14).

A two-state ‘gated scanner' model can account for these and previous observations of events that occur during Chi recognition (14), including the allosteric coupling we observe between the Chi binding locus and the integrity/position of the latch (Figure 8). In the absence of Chi sequences, the equilibrium position of the latch is closed, as observed in all crystal structures of the AddAB complex so far. This ‘AddAB state’ is stabilized by interactions that include the conserved salt bridge between E129 and R629. The closed gate guides 3′-terminated DNA from the helicase motor towards the Chi scanner and then past the nuclease domain, where it is occasionally cleaved as it leaves via the primary exit channel. The 5′-terminated strand is also cleaved and so AddAB moves along DNA operating in a *destructive mode*, degrading both nascent strands of the duplex. During translocation, the scanner binds DNA to test for Chi sequences (Figure 8, step 1), with the lifetime of these events determined by the similarity of the sequence to the canonical Chi pentamer. This transient form, which we term the *stalled encounter complex*, is responsible for the pausing observed in single molecule experiments. There is no conformational change, and so the AddAB is stationary because DNA can no longer move through the enzyme complex to leave via the primary exit channel. We suggest that a recently-solved structure of AddAB bound to Chi resembles this state, and that it reveals why the helicase motor domains are unable to drive the enzyme forward (23). Translocation can restart in two possible ways. Firstly, the rapid unbinding of the DNA from the scanner is favoured when it does not contain a *bona fide* Chi sequence, thereby avoiding mis-recognition events (Figure 8, step 2). Again, there is no conformational change and the enzyme returns to the *destructive mode*. Alternatively, the enzyme may undergo a conformational change into the ‘AddAB* state', thereby transforming into the *recombinogenic mode* associated with nuclease attenuation, translocation rate change and DNA unwinding stimulation (Figure 8, step 3). This state is elicited if the latch opens while a DNA sequence (i.e. Chi) remains engaged with the scanner, thereby opening the gate to an alternative exit channel and allowing extrusion of a ssDNA loop. This path would be favoured for *bona fide* Chi sequences if their higher binding energy were harnessed to drive structural rearrangements that open the latch. The structural basis for this allosteric coupling is not understood, but might simply involve a concerted conformational change at the latch and the Chi binding locus as the enzyme fully engages with the hotspot sequence (as indicated conceptually in Figure 8). This idea is feasible and attractive, because a structure of AddAB in an initial encounter complex with Chi (23), shows that the latch is connected to the Chi binding locus: a recognition helix that interacts with the phosphate group immediately to the 5′-side of Chi via R132, also forms a salt bridge to the latch via E129 (Figure 5). Alternatively, or additionally, the allostery might be less conventional and involve the ‘pushing’ of the DNA motor domain against the stable AddAB:Chi interaction to force the opening of the latch. A full understanding of the structural transition to the AddAB* state will ultimately await further structural information.

Our model can also explain the behavior of mutant complexes. In the AddAB^E129A^ enzyme the closed position of the latch is destabilized, shifting the equilibrium position towards the open AddAB* state. This would result in more efficient recognition of Chi (Figure 8, step 3), but this is at the expense of the promiscuous ‘recognition' of non-specific DNA (Figure 8, step 4 and *promiscuous recombinogenic mode*), both of which we have observed experimentally here. As would be expected from the model, there is no decrease to the pausing frequency of AddAB in the latch mutant (this work), whereas mutations to the Chi binding site eliminate pausing at both Chi and Chi-like sequences (14), presumably by destabilizing the Chi encounter complex and increasing the rate of Chi unbinding (Figure 8, arrow 2). It is perhaps surprising that the increased stability of the AddAB*:Chi complex that is afforded by the latch mutation (∼15-fold) is much greater than the increase in apparent Chi recognition efficiency (∼2-fold). However, the initial recognition of Chi to form an encounter complex is a complicated molecular event because it competes with DNA translocation. Moreover, the subsequent extrusion of a ssDNA loop through an alternate exit to form the mature AddAB:Chi complex might very well change the manner in which AddAB is able to dissociate from DNA. The recognition of Chi by AddAB is also modulated by the binding of ATP to a second site in AddB protein. Interestingly, mutations that would be expected to prevent ATP binding at this site significantly reduce the stability of the Chi complex, with no measurable effect at all on the Chi recognition efficiency measured in attenuation assays (6).

The identification of the latch as being a conserved and important structural feature in both AddAB- and RecBCD-type enzymes provides a framework to examine the more enigmatic AdnAB class of helicase-nucleases found in mycobacteria and streptomycetes. It is not known whether AdnAB is regulated by Chi (3,28). However, based on the sequence analysis performed in (28), there is an appropriate insertion in the AdnA (∼AddB) subunit that is predicted to fold into an alpha helix (data not shown). It is therefore possible that AdnAB does contain a latch-like structure, implying a regulatory mechanism of some form. Validation of this hypothesis will however await further structural and mechanistic studies of the AdnAB-type enzymes.

The absolute conservation of the latch structure in AddAB and RecBCD emphasizes the importance of quality control in Chi sequence recognition. This mechanism creates an energetic barrier that acts as a selectivity filter for *bona fide* Chi sequences, such that recognition efficiency is sacrificed in favour of sequence selectivity. In turn, this raises the question of why DNA break resection is regulated by Chi in bacterial cells, when there is no suggestion of this complexity in other domains of life (1). Modulation of resection by Chi sequences certainly helps to optimize the resection product for recombination, by producing a 3’-terminal OH in a G-rich sequence which is ideal for subsequent priming of replication, and promoting stable DNA unwinding and the loading of RecA protein onto the ssDNA. Moreover, the over-representation of Chi sequences in many bacterial genomes enables them to act in a self-recognition capacity, ensuring that phage and other foreign DNAs are more likely to be degraded, whereas self-DNA is targeted for repair. This anti-phage role has recently been re-enforced by the exciting observation that the fragments of DNA formed upstream of Chi are fed to the Cas1/2 complex for incorporation into CRISPR libraries (29). It appears that there is still more to learn about the important role of Chi recombination hotspots in bacterial evolution.

## Supplementary Material

SUPPLEMENTARY DATA
