# Supplementary material for: Chi hotspots trigger a conformational change in the helicase-like domain of AddAB to activate homologous recombination
Source: Nucleic Acids Res. 2016 Jan 13;44(6):2727–41. doi: 10.1093/nar/gkv1543 (PMC4824097; doi:10.1093/nar/gkv1543)
Supplement: SUPPLEMENTARY DATA [file supp_44_6_2727__index.html]

Chi hotspots trigger a conformational change in the helicase-like domain of AddAB to activate homologous recombination — Chi hotspots trigger a conformational change in the helicase-like domain of AddAB to activate homologous recombination — SUPPLEMENTARY DATA 

# Chi hotspots trigger a conformational change in the helicase-like domain of AddAB to activate homologous recombination

## SUPPLEMENTARY DATA

- SUPPLEMENTARY DATA
