# Supplementary material for: Chi hotspots trigger a conformational change in the helicase-like domain of AddAB to activate homologous recombination
Source: Nucleic Acids Res. 2016 Jan 13;44(6):2727–41. doi: 10.1093/nar/gkv1543 (PMC4824097; doi:10.1093/nar/gkv1543)

Chi hotspots trigger a conformational change in the helicase-like domain of AddAB to activate homologous recombination

Neville S. Gilhooly, Carolina Carrasco, Benjamin Gollnick, Martin Wilkinson, Dale B. Wigley, Fernando Moreno-Herrero, and Mark S. Dillingham

Supplementary Information

Supplementary Methods

Supplementary Discussion 1-3

Supplementary Figures 1-8

Supplementary References

Supplementary Methods

Mass spectrometry

Analysis of the 110 kDa band of interest was performed by the University of Bristol Proteomics service. Gel bands were subjected to in-gel tryptic digestion using a DigestPro automated digestion unit (Intavis Ltd.). The resulting peptides were diluted 1000-fold and fractionated using an Ultimate 3000 nanoHPLC system in line with an LTQ-Orbitrap Velos mass spectrometer (Thermo Scientific). In brief, peptides in 1% (vol/vol) formic acid were injected onto an Acclaim PepMap C18 nano-trap column (Thermo Scientific). After washing with 0.5% (vol/vol) acetonitrile 0.1% (vol/vol) formic acid peptides were resolved on a 250 mm × 75 μm Acclaim PepMap C18 reverse phase analytical column (Thermo Scientific) over a 150 min organic gradient, using 7 gradient segments (1-6% solvent B over 1min., 6-15% B over 58min., 15-32%B over 58min., 32-40%B over 3min., 40-90%B over 1min., held at 90%B for 6min and then reduced to 1%B over 1min.) with a flow rate of 300 nl min^{-1}. Solvent A was 0.1% formic acid and Solvent B was aqueous 80% acetonitrile in 0.1% formic acid. Peptides were ionized by nano-electrospray ionization at 2.1 kV using a stainless steel emitter with an internal diameter of 30 μm (Thermo Scientific) and a capillary temperature of 250°C. Tandem mass spectra were acquired using an LTQ- Orbitrap Velos mass spectrometer controlled by Xcalibur 2.1 software (Thermo Scientific) and operated in data-dependent acquisition mode. The Orbitrap was set to analyze the survey scans at 60,000 resolution (at m/z 400) in the mass range m/z 300 to 2000 and the top twenty multiply charged ions in each duty cycle selected for MS/MS in the LTQ linear ion trap. Charge state filtering, where unassigned precursor ions were not selected for fragmentation, and dynamic exclusion (repeat count, 1; repeat duration, 30s; exclusion list size, 500) were used. Fragmentation conditions in the LTQ were as follows: normalized collision energy, 40%; activation q, 0.25; activation time 10ms; and minimum ion selection intensity, 500 counts.

The raw data files were processed and quantified using Proteome Discoverer software v1.2 (Thermo Scientific) and searched against a combined database consisting of the UniProt *Bacillus subtilis* 168 database (4217 sequences) plus the AddA and AddB sequences using the SEQUEST algorithm. Peptide precursor mass tolerance was set at 10ppm, and MS/MS tolerance was set at 0.8Da. Search criteria included carbamidomethylation of cysteine (+57.0214) as a fixed modification and oxidation of methionine (+15.9949) as a variable modification. Searches were performed with full tryptic digestion and a maximum of 1 missed cleavage was allowed. The reverse database search option was enabled and all peptide data was filtered to satisfy false discovery rate (FDR) of 5%.

Dye displacement helicase assay

Real-time helicase measurements were performed and analysed as described previously (1). Experiments were performed at 37°C in a stopped-flow instrument with a xenon-mercury light source (TgK Scientific). Hoechst 33258 dye was excited at 366 nm through 1 mm slits, and the fluorescence above 400 nm was subsequently recorded. DNA molecules (0.1 nM) were incubated with 1 nM streptavidin in a buffer containing Hoechst 33258 (200 nM), SSB_4 protein (200 nM), BSA (100 mg/ml), tris acetate (25 mM, pH 7.5), magnesium acetate (2 mM) and DTT (1 mM). AddAB enzymes (1 nM), were added to the preformed DNA:streptavidin complexes and incubated at 37°C for 2 minutes prior to mixing against an equal volume of the same solution but with the streptavidin and DNA substrate omitted and $AddA^{K36A}B$ enzymes (30 nM) and ATP (0.5 mM) added. All concentrations stated are post-mixing. The use of the $AddA^{K36A}B$ mutant prevents re-binding of the enzyme being studied ensuring single-turnover conditions with respect to the DNA substrate (1).

Limited proteolysis with crystallographic substrates

Limited proteolysis experiments were conducted essentially as described in the main text but using crystallographic hairpin DNA substrates with unpaired single-stranded tails. Two substrates were used; Hairpin-Chi+ included a Chi sequence in the correct position for immediate recognition whereas in Hairpin-Chi0 the hotspot sequence was replaced by five thymidine bases (see table below). A single base was included 3' of Chi to mimic the cleaved product post Chi-recognition. In advance of the reaction, substrates were annealed and purified as described previously (2). Mutant AddAB enzymes were expressed and purified as described previously (3). The latch mutants were made in combination with a double nuclease inactivation mutant (D1172A in AddA and D961A in AddB) to mimic the crystallographic conditions that prevent digestion of the exposed single-strand tails. AddAB was pre-incubated with excess DNA in standard reaction buffer at 37°C for 10 minutes. The processing of DNA substrates was initiated by the addition of ATP to a concentration of 1 mM. After 20 seconds, α-Chymotrypsin was added to a concentration of 0.5 μg/ml. 10 μL aliquots were removed at indicated time points and added to 10 μL of stop buffer and immediately placed at 95°C for 2 minutes prior to electrophoresis.

Oligonucleotides used in this study

Oligo name	Sequence (5´>3´)
5-Chi tail<br>(Chi in bold)	TTTTTTTTTTTTTTTTTTTTTTTTTTATCTT**AGCGG**TCTT**AGCGG**TAT**AGCGG**TGTT**AGCGG**TTC**AGCGG**ATT
5- Chi_2	AATCCGCTGAACCGCTAACACCGCTATACCGCTAAGACCGCTAAGATA
5-Chi Scramble	TTTTTTTTTTTTTTTTTTTTTTTTTTATCTTGCGAGTCTTGCGAGTATGCGAGTGTTGCGAGTTCGCGAGATT
5-Chi Scramble_2	AATCTCGCGAACTCGCAACACTCGCATACTCGCAAGACTCGCAAGATA
Hairpin-Chi+<br>(Chi in bold)	TTTTTTTCTAATGCGAGCACTGCTATTCCCTAGCAGTGCTCGCATTAGATTTTGTTTTTTT**AGCGG**T
Hairpin-Chi0	TTTTTTTCTAATGCGAGCACTGCTATTCCCTAGCAGTGCTCGCATTAGATTTTGTTTTTTTTTTTTT
TFO	TAM - TTCTTTTCTTTCTTCTTTCTTT

Supplementary Discussion

Supplementary discussion 1 - Chi is recognised in the context of small oligonucleotide based DNA substrates.

Break resection reactions were conducted to confirm that the substrates used in proteolysis experiments behaved similarly to more conventional long substrates with respect to nuclease regulation by Chi. The experiments were performed in standard buffer conditions (see main text), but in the absence of SSB protein, and were run on 8% denaturing polyacrylamide gels to resolve the short resection products.

The wild type enzyme processes the Chi-containing 73 mer to produce a series of bands that run below the full length substrate DNA (**SFigure 3**). If the enzyme cuts predominantly 1 ntd upstream of Chi as expected (4), then the predicted Chi-dependent cleavage products should be 71, 63, 54, 46 and 37 nucleotides long. When looking at the lane profile of this reaction distinct peaks occur at these predicted cleavage positions. Furthermore, the Chi recognition mutant $AddAB^{F210A}$ produces the same banding pattern, but these bands are all less intense than with the wild-type (6% vs 17% of total input DNA). This indicates that these bands reflect cleavages made in the DNA in response to Chi. When Chi is absent in the "scrambled" control substrate, there are no ATP-dependent bands that run below the substrate. AddAB has presumably unwound the DNA without degrading it because the lifetime of the unwinding event is much smaller than the time required to cleave the DNA. This is as expected based on the infrequent cleavage pattern observed on much longer substrates (5).

Supplementary discussion 2 - The proteolysis product of AddAB* maps to the helicase-like domain of AddB and close to the latch binding pocket.

Evidence for a conformational change upon Chi recognition was provided by a change in the products of limited proteolysis. To gain further structural insight into the nature of this change, we initially attempted to map the Chi-dependent proteolytic site using N-terminal sequencing, but this repeatedly failed due to a lack of material. We subsequently turned to Orbitrap mass spectrometry to identify the peptide products from *in situ* tryptic digests of the band of interest. This allows us to crudely reconstruct the domain boundaries of the band of interest by scoring the positions of the peptide hits. We performed the experiment on both the wild type and latch mutant complexes and, as a control, we also analysed an equivalent sized gel piece from the same position in the lane of a "scrambled" DNA experiment. Due to the exquisite sensitivity of this method, we deliberately diluted both the samples and the controls to obtain a very low coverage of either AddA or AddB in the control sample (see Mass Spectrometry Methods).

Under the identical conditions for the experimental samples, we obtained very low coverage of AddA, but consistently obtained peptides that covered much of the C-terminal region of AddB. The peptides mapped from as early as residue 265 to near the C-terminus. Removal of 265 residues from the N-terminus produces a protein of theoretical mass 103 kDa, which is within error of our estimate for the molecular mass of the proteolysis product (~110 kDa) from molecular weight markers. The possible cleavage region is within the helicase-like domain of AddB and includes the latch binding pocket. The results are therefore consistent with, but do not prove, movement of the ionic latch structure upon Chi recognition.

Supplementary discussion 3 – The latch mutant displays similar biochemical properties to Chi-modified wild type AddAB.

The modification of AddAB by Chi can be monitored by at least three biochemical hallmarks: attenuation of 3′-directed nuclease activity (4), reduction in translocation rate (6,7) and stimulation of helicase activity at limiting [SSB] (1). Experiments in the main paper using triplex displacement assays show that the latch mutant has translocation properties that partially resemble the Chi-modified wild type even in the absence of Chi sequences. To corroborate the triplex displacement results we measured helicase activity in real-time as described in (1) (**SFigure 8**). In agreement with our published conclusions that Chi recognition stimulates DNA strand separation, we observe a dose-dependent increase in the rate and extent of DNA unwinding that occurs precisely when wild type AddAB reaches the Chi sequence (~0.3 s). The observed unwinding amplitudes can be plotted against the number of Chi sequences and fit to a simple model of Chi recognition (1) to obtain an apparent probability of recognising Chi of 0.31 for wild type AddAB (black curve, **SFigure 8c**). The behaviour of the latch mutant is qualitatively similar, in that DNA unwinding is stimulated precisely at Chi. However, the extent to which DNA unwinding is stimulated is greater for the latch mutant than the wild-type, consistent with the improved Chi recognition associated with this enzyme. The fit to the data returns an increased probability of Chi recognition of 0.52 for AddAB^{E129A}. Additionally, these data reveal that the latch mutant can unwind Chi-free DNA better than the wild-type (23% versus 13%, **SFigure 8c**). A simple interpretation of this result that is consistent with the triplex data is that the latch mutant can spontaneously convert to the AddAB* form on Chi-free DNA.

<u>Supplementary Figure legends</u>

Supplementary Figure 1 – Channels for 3′- and 5′-terminated ssDNA strands in the AddAB complex.

A transparent surface of the AddAB complex (PDB: 4CEJ) is shown with AddA and AddB in red and grey respectively. The N-terminal helicase-like domain of AddB is also shown in grey cartoon format, with the latch helix highlighted in blue. A DNA substrate (black cartoon) is shown bound to the enzyme, with the duplex at the front, and ssDNA extending through separate channels to exits at the top and rear. Dotted lines show the expected path of the nascent DNA where the crystallographic substrate does not extend fully through the channels. In this structure the 5 bases of the Chi sequence (yellow) are bound to AddB, such that the 3′-terminated DNA can no longer leave the complex via the primary exit channel. A channel bypass model (8) hypothesises that continued translocation of AddAB will be facilitated by movement of the latch helix to open an alternative exit channel on the underside of the enzyme.

Supplementary Figure 2 - Biochemical properties of AddAB complexes containing mutations in both the Chi recognition locus and the ionic latch.

(a) Double mutants resect Chi-free DNA normally. 1.6 nM 5′-radiolabelled and linearised Chi-free plasmids were processed by 0, 0.5, 1, 2 and 4 nM AddAB enzymes for 4 minutes at 37 ^{o}C in the presence of 2 μM SSB and 1 mM ATP. A control with 2 nM wild-type AddAB is shown in the lane on the far right (M). Black triangles represent the linear DNA substrate, blue triangles show the position of full length ssDNA. The gels were uniformly contrast enhanced to allow visualisation of resection products. (b) $AddAB^{F68A+E129A}$ forms more stable complexes with Chi than $AddAB^{F68A}$. Exonuclease I chase of Chi fragments formed by wild-type (WT) and E129A. Lane S represents the unprocessed substrate DNA and lane P represents the products of the reaction prior to the addition of the exonuclease chase. The yield of Chi fragment in lane P is taken as an estimate of the initial amount of Chi fragment. Subsequent time points are relative to the time that the exonuclease was added. 1.6 nM DNA molecules were processed by 4 nM AddAB enzymes in the presence of 2 μM SSB and 1 mM ATP at 37 ^{o}C. ExoI is added to a final concentration of 0.8 units/μl at 30 seconds. Black triangles represent the tailed DNA substrate, blue triangles show full length ssDNA, red triangles indicate the Chi fragment and green triangles show the primosome assembly site (PAS), a region of secondary stucture that is ExoI resistant. The gels have been contrast enhanced to aid visualisation of reaction products. (c) Quantification of total Chi fragment as a

function of time. Both data sets were fit to a single exponential decay to zero. The half life ($t_{½}$) is indicated and is about 15-fold greater for the double mutant complex.

Supplementary Figure 3 – Chi is recognised in the context of small oligonucleotide-based DNA substrates.

(a) Schematic of the DNA substrates used in the proteolysis experiments. The Chi-containing substrate has five correctly oriented Chi sequences, whereas the control substrate has the same overall sequence content but zero Chi sequences. See table above for the sequences of the oligonucleotides used. (b) DNA substrates (1 nM) were pre-bound to 4 nM AddAB enzymes at 37°C before the addition of ATP (1 mM) to initiate the reaction. After 30 seconds the sampled were boiled and electrophoresed through an 8% TBE-urea sequencing gel. Experiments were performed either with wild type (WT) or Chi binding mutant (AddAB^{F210A}, MT) complex. Grey arrows indicate the positions of Chi fragments. (c) Lane profiles of wild-type (red) and Chi binding mutant (blue) for the Chi DNA substrates. The marker positions are indicated in nucleotides (small black arrows). The Chi-specific cleavage fragments are indicated with larger grey arrows.

Supplementary Figure 4 - The Chi-dependent proteolysis product is ATP- and Chymotrypsin-dependent, is not associated with a difference in Fe-S cluster occupancy between preparations, is formed using DNA substrates employed in crystallography, and is potentiated by both "latch mutations" AddB E129A and R629A.

(a) Proteolysis experiments were performed as in Figure 4 in the main text, except that single time points were taken at 4 minutes. Experiments were performed with various components omitted (indicated) to test the DNA, ATP, Chi and Chymotrypsin dependence of the formation of the ~110 kDa band of interest. Note that this faint band only appears in lane 5 and is increased in lane 8 when the latch mutant complex is used. Lower panel shows a zoomed-in version of the gel focusing on the area marked with a black bar. The band of interest is highlighted with a red asterisk. (b) Proteolysis reactions were conducted with Chi-containing DNA substrates and two fixed timepoints (indicated). The AddAB^{C1130} complex is unable to coordinate an Fe-S cluster and is sensitive to proteolytic degradation (9). This enzyme serves as a control to show that the band of interest is not a proteolysis product of AddAB that has lost the Fe-S cluster. Experiments with wild type, latch mutant and Chi-binding mutant complexes are also shown. Lower panel shows a zoomed-in version of the gel focusing on the area marked with a black bar. The band of

interest is highlighted with a red asterisk. (c) Experiments were designed to test whether the AddAB complexes and substrates we have used in crystallographic studies of AddAB support the Chi-dependent conformational change (see **Supplementary Methods** for details). All experiments use a double-nuclease mutant in addition to the mutations designed to destabilise the latch. (i) Schematic of the hairpin oligonucleotides used in these experiments. (ii) The 110kDa proteolysis product (*) is formed in a Chi-dependent manner. These experiments were performed with the AddAB^{E129A} complex with α-Chymotrypsin incubations of 2, 4 and 6 minutes respectively. (iii) The formation of the 110kDa Chi-dependent product is potentiated by both the E129A mutation in AddB domain 1B, and the R629A mutation in the latch itself. Reactions were carried out with the Chi substrate and the two α-Chymotrypsin incubation times used were 2 and 5 minutes respectively. The band of interest is presumably produced in very low yield in experiments without mutation of the latch because only a single Chi sequence is present in these hairpin substrates (five are used in the experiments shown in the main text).

Supplementary Figure 5 – The Chi-dependent proteolysis product is the C-terminus of AddB.

(a) Example of gels used for analysis of the ~110 kDa band of interest that is formed by proteolysis of AddAB following processing of Chi-containing DNA substrates. Experiments were performed with either the wild type or latch mutant AddAB complex and using either Chi-containing (Chi) or Chi-free (Ctr) DNA substrates. As demonstrated in the main paper, the band of interest is only observed following processing of Chi-containing substrates and the yield is considerably larger for the latch mutant complex. The bands of interest (or equivalently-positioned gel slices for the control experiments) were excised, subjected to *in situ* tryptic digestion, and analysed by mass spectrometry. (b) Mass spectrometry results. Number of peptides detected and ion score for the AddA and AddB polypeptides in each experiment (WT, wild type; MT, latch mutant). The ion score can be used as a measure of relative quantification of the same protein between experiments. The AddB polypeptide is confidently identified in both experiments containing the Chi DNA, but not in the control experiment. The AddA polypeptide is never confidently identified. Indeed, the values obtained for AddA (and for AddB in the control experiment) are comparable to those of several other *B. subtilis* proteins which must either be low level contaminants of AddAB preparations, or arise as the result of false discoveries (data not shown). (c) The positions of the identified peptides within AddB for the wild type and latch mutant experiment. Green and yellow bars indicate high (>99%) and medium (>95%) detection confidence respectively. In both cases, there is good coverage for most of the C-terminal region, and no coverage at all of the first ~300 amino acids. The transparent red rectangle indicates the range of single cleavage positions that could give rise to a 110 kDa

polypeptide assuming an error of +/-10 kDa. Note that the position of the square is in good agreement with the start of the peptide coverage in the C-terminus.

Supplementary Figure 6 – Single molecule DNA translocation assays

(a) Cartoon representation of the DNA substrates used in magnetic tweezers experiments. Representative single molecule translocation traces at 3 Hz are shown for (b) wild-type and (c) latch mutant AddAB measured at 37°C. (d) Mean velocity as a function of position on DNA for wild-type and latch mutant AddAB measured at the temperatures indicated. Each curve corresponds to the average of at least 40 individual velocity traces. The sharp dip observed in the wild type AddAB translocation rate curve that occurs between 4 and 5 kbp is due to Chi sequence recognition. The latch mutant pause at 37 ºC is most likely too short to be resolved. (e) Location and frequency of pauses for wild-type and latch mutant AddAB. Pausing at Chi was hardly detected and pausing outside of the Chi locus was similar for both wild type and latch mutant AddAB.

Supplementary Figure 7 – Triplex displacement assays.

(a) Schematic of substrates used in triplex displacement experiments to test the effect of Chi recognition on DNA translocation. The positions (in base pairs) of a Chi locus containing between zero and three Chi sequences, and of the triplex (magenta bar) are indicated. The distal end of the substrate is blocked by a biotin-streptavidin complex to ensure unidirectional translocation. (b) Triplex displacement curves on the substrates shown in (a) using either wild type (faded grey) or latch mutant (coloured) AddAB complexes. These data are reproduced from the main paper for comparison with panels (c) and (d) which show the same experiment performed with the double mutant complexes AddAB$^{F210A+E129A}$ and AddAB$^{F68A+E129A}$, respectively. Note that the 1st phase amplitude is similar to the single latch mutant complex in both cases, and reduced compared to wild type. (e) Direct comparison of the absolute amplitudes of triplex displacement for wild type and latch mutant AddAB on a Chi-free DNA substrate, showing that the two proteins have similar processivity. (f) Direct comparison of the absolute amplitudes of triplex displacement for wild type and latch mutant AddAB on a DNA substrate containing three Chi sequences. The substrates used in panels (e) and (f) are as shown in the main Figure 7a, and the data is equivalent to some of that found in main Figure 7b, with the exception that it has not been normalized to percent of maximum fluorescence.

Supplementary Figure 8 – Real time helicase assays.

(a) Schematic of the substrates used in stopped-flow DNA unwinding assays. These contain variable numbers of Chi sequences positioned at a locus ~500 bp from the DNA end entry point. (b) DNA unwinding as a function of time for wild type and latch mutant AddAB. The traces are colour coded according to the DNA substrate key. The kinetics of unwinding are strongly stimulated upon arrival at Chi sequences in a dose-dependent manner. (c) Quantification of the final amplitude of unwinding for different numbers of Chi sequences, from the data shown in panel (b).

Supplementary References

1. Yeeles, J.T., van Aelst, K., Dillingham, M.S. and Moreno-Herrero, F. (2011) Recombination Hotspots and Single-Stranded DNA Binding Proteins Couple DNA Translocation to DNA Unwinding by the AddAB Helicase-Nuclease. *Mol Cell*, **42**, 806-816.
2. Singleton, M.R., Dillingham, M.S., Gaudier, M., Kowalczykowski, S.C. and Wigley, D.B. (2004) Crystal structure of RecBCD enzyme reveals a machine for processing DNA breaks. *Nature*, **432**, 187-193.
3. Krajewski, W.W., Fu, X., Wilkinson, M., Cronin, N.B., Dillingham, M.S. and Wigley, D.B. (2014) Structural basis for translocation by AddAB helicase-nuclease and its arrest at chi sites. *Nature*, **508**, 416-419.
4. Chedin, F., Ehrlich, S.D. and Kowalczykowski, S.C. (2000) The Bacillus subtilis AddAB helicase/nuclease is regulated by its cognate Chi sequence in vitro. *Journal of molecular biology*, **298**, 7-20.
5. Yeeles, J.T. and Dillingham, M.S. (2007) A dual-nuclease mechanism for DNA break processing by AddAB-type helicase-nucleases. *Journal of molecular biology*, **371**, 66-78.
6. Carrasco, C., Gilhooly, N.S., Dillingham, M.S. and Moreno-Herrero, F. (2013) On the mechanism of recombination hotspot scanning during double-stranded DNA break resection. *Proc Natl Acad Sci U S A*.
7. Gilhooly, N.S. and Dillingham, M.S. (2014) Recombination hotspots attenuate the coupled ATPase and translocase activities of an AddAB-type helicase-nuclease. *Nucleic acids research*.
8. Yang, L., Handa, N., Liu, B., Dillingham, M.S., Wigley, D.B. and Kowalczykowski, S.C. (2012) Alteration of chi recognition by RecBCD reveals a regulated molecular latch and suggests a channel-bypass mechanism for biological control. *Proceedings of the National Academy of Sciences of the United States of America*, **109**, 8907-8912.
9. Yeeles, J.T., Cammack, R. and Dillingham, M.S. (2009) An iron-sulfur cluster is essential for the binding of broken DNA by AddAB-type helicase-nucleases. *J Biol Chem*, **284**, 7746-7755.

Gilhooly et al, SFigure 1

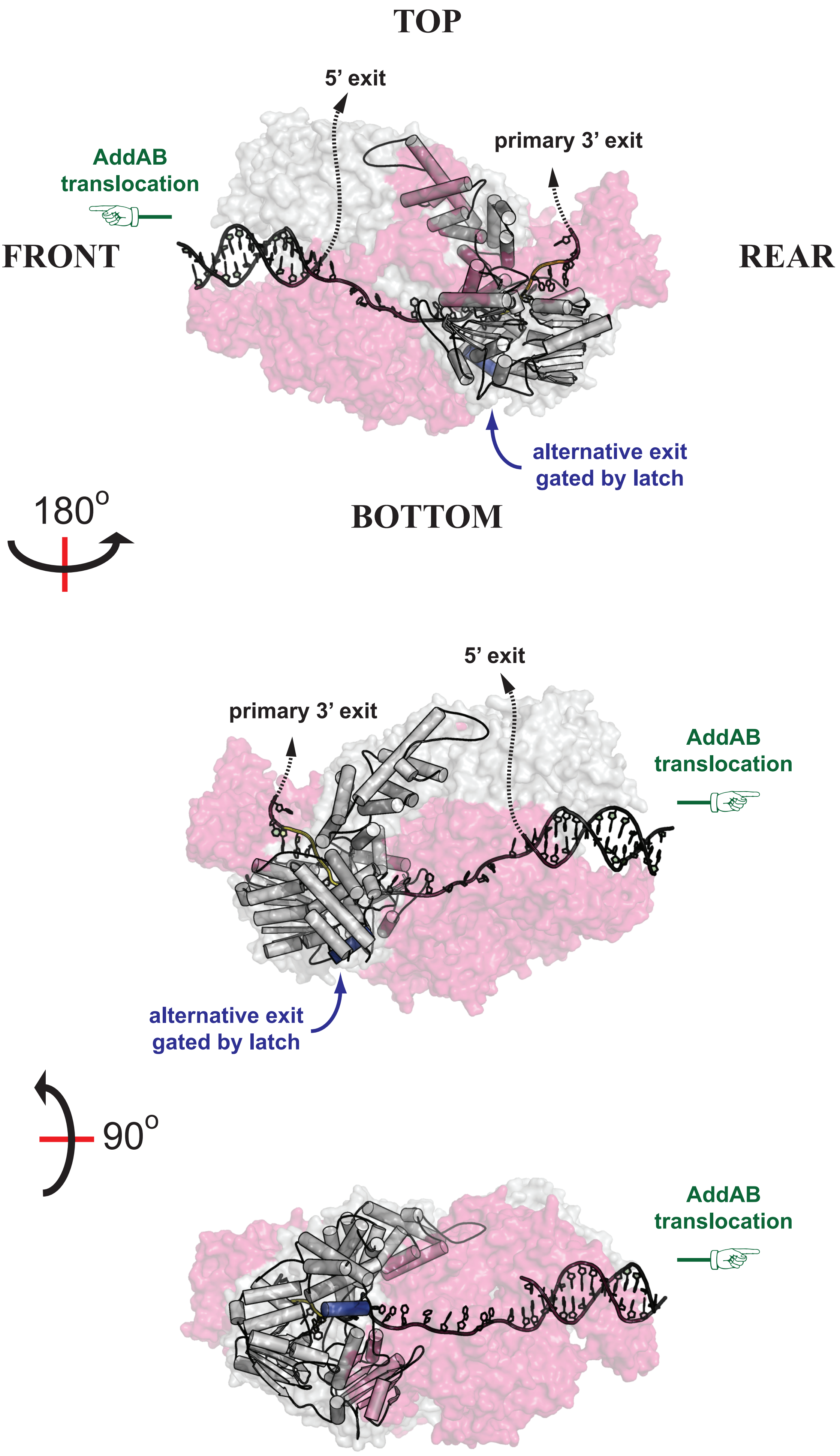

Gilhooly et al, SFigure 2

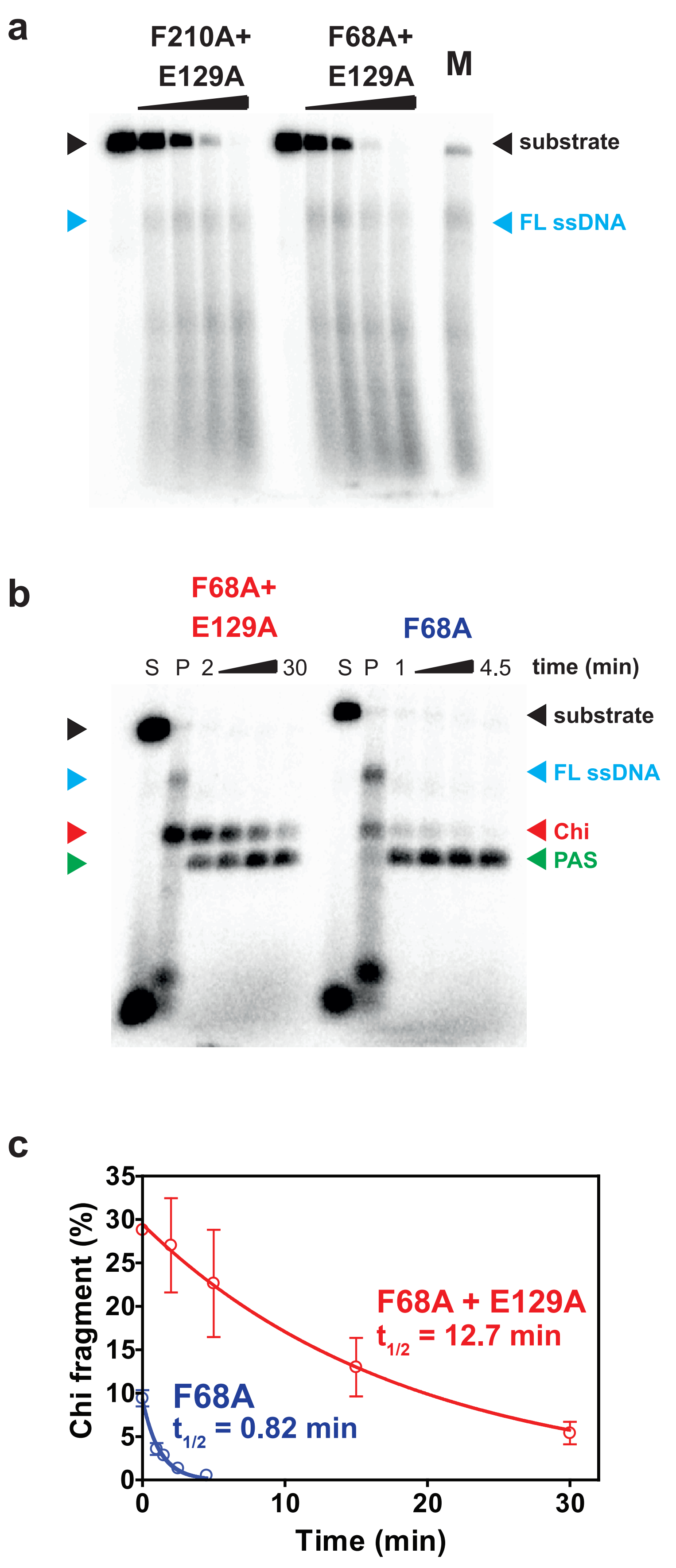

Gilhooly et al, SFigure 3

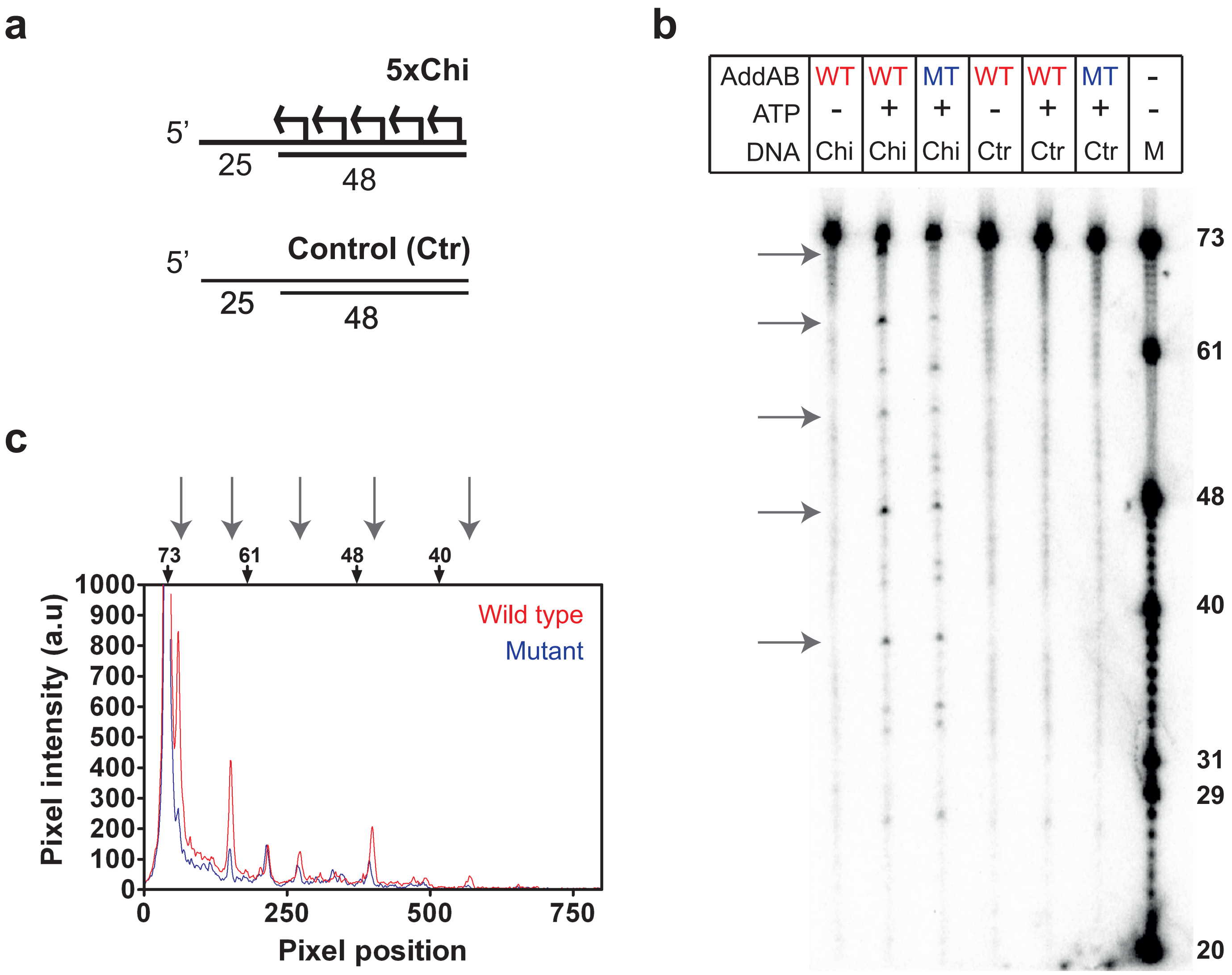

Gilhooly et al, SFigure 4

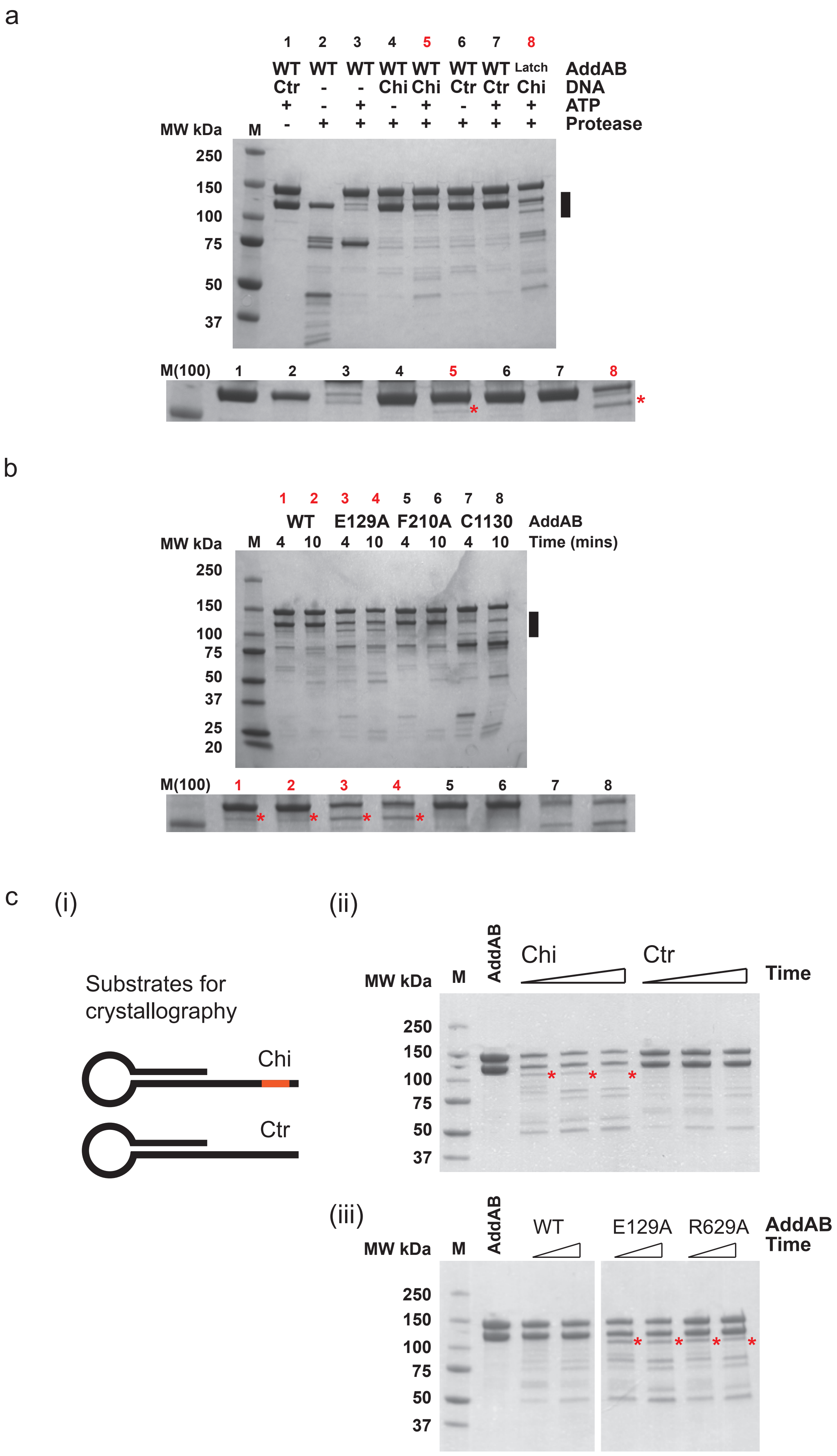

Gilhooly et al, SFigure 5

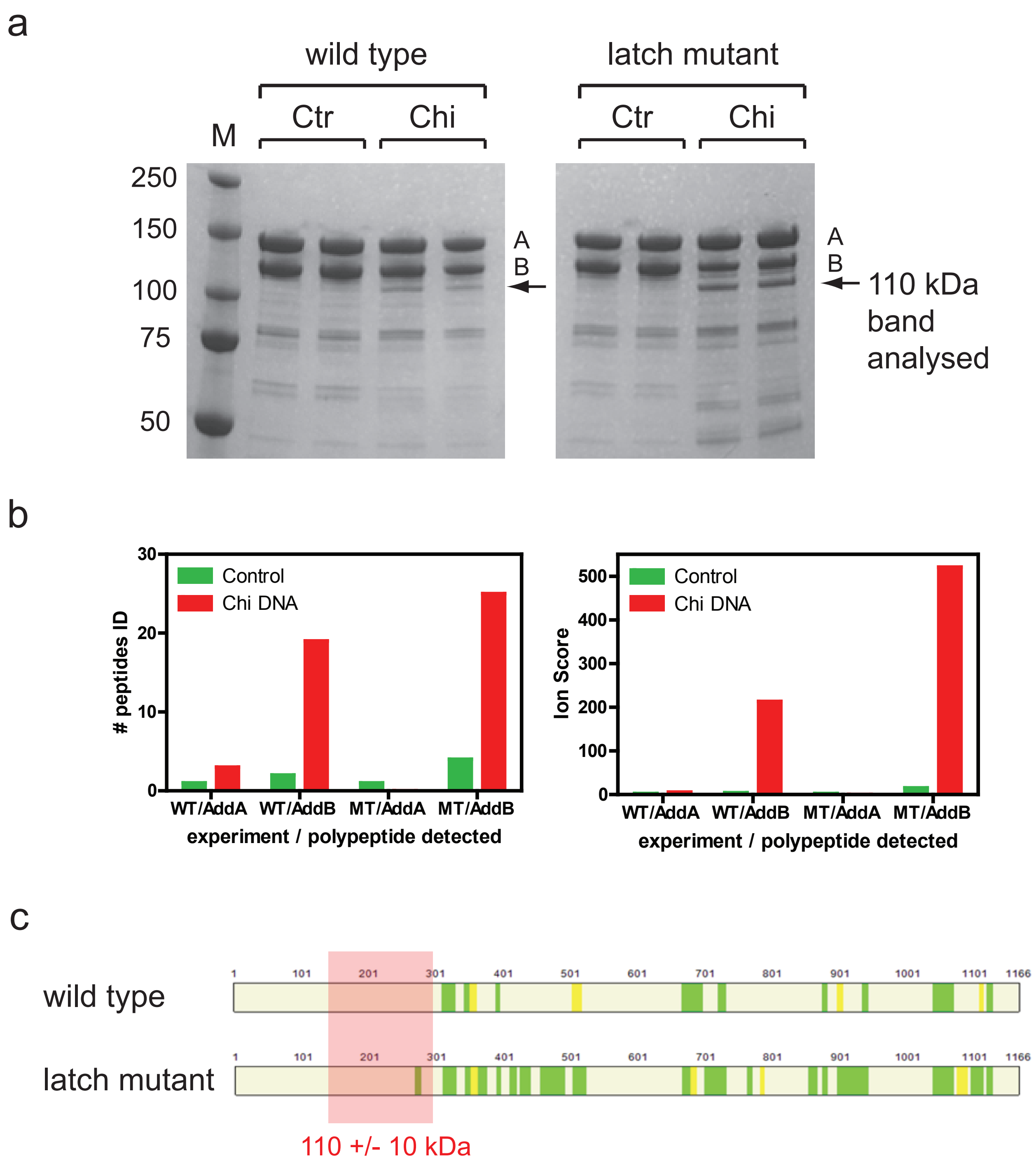

Gilhooly et al, SFigure 6

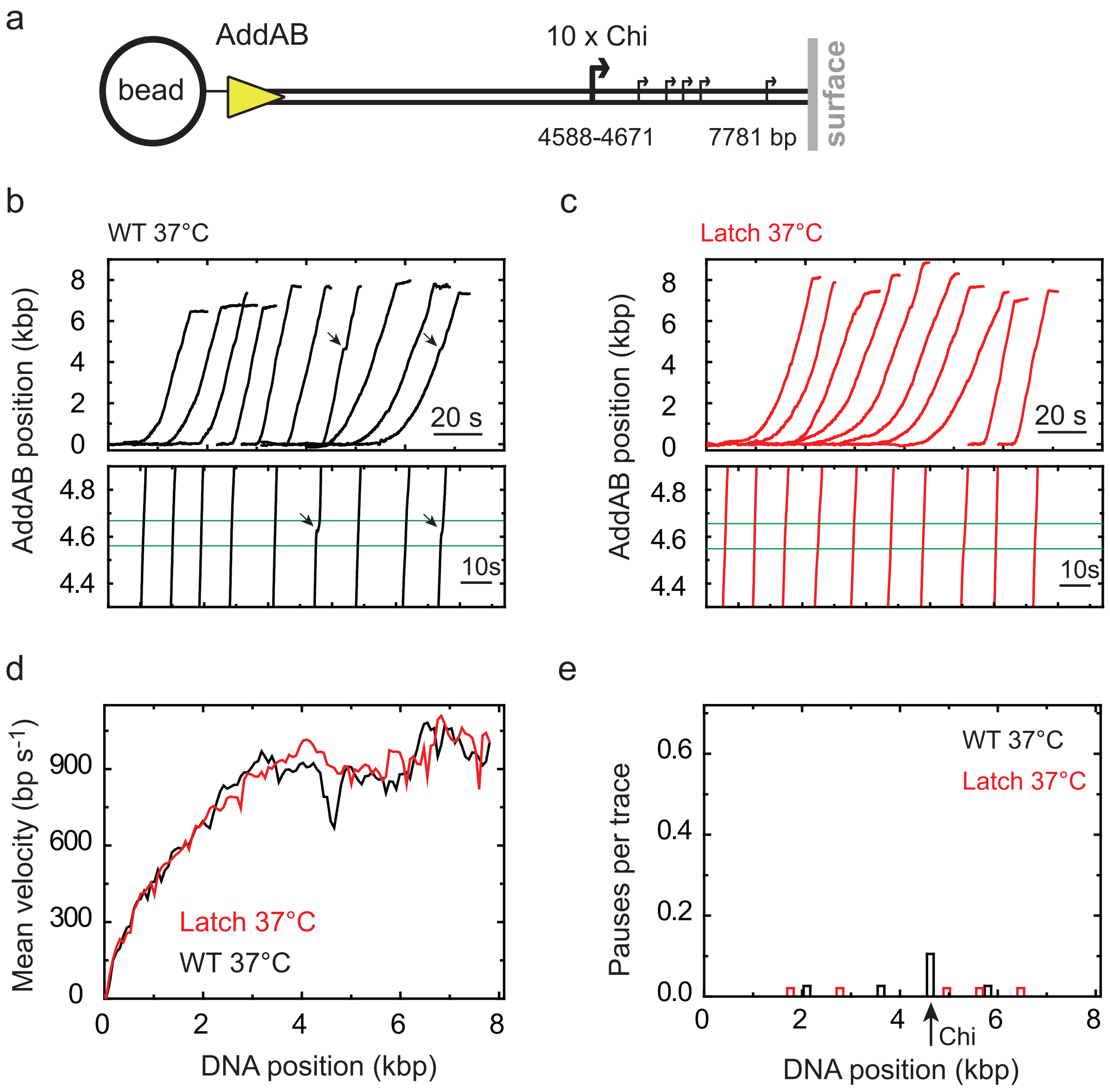

Gilhooly et al, SFigure 7

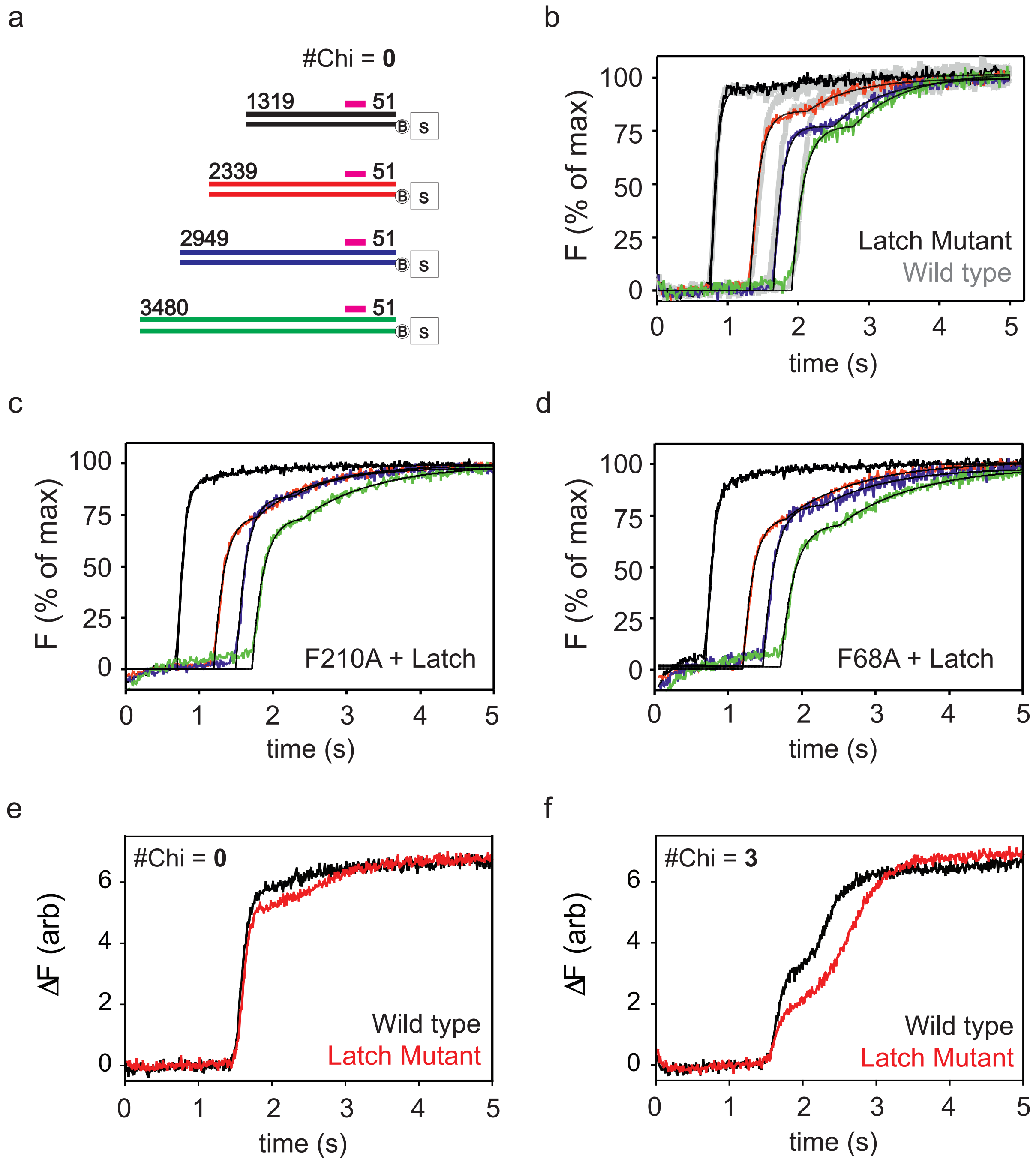

Gilhooly et al, SFigure 8

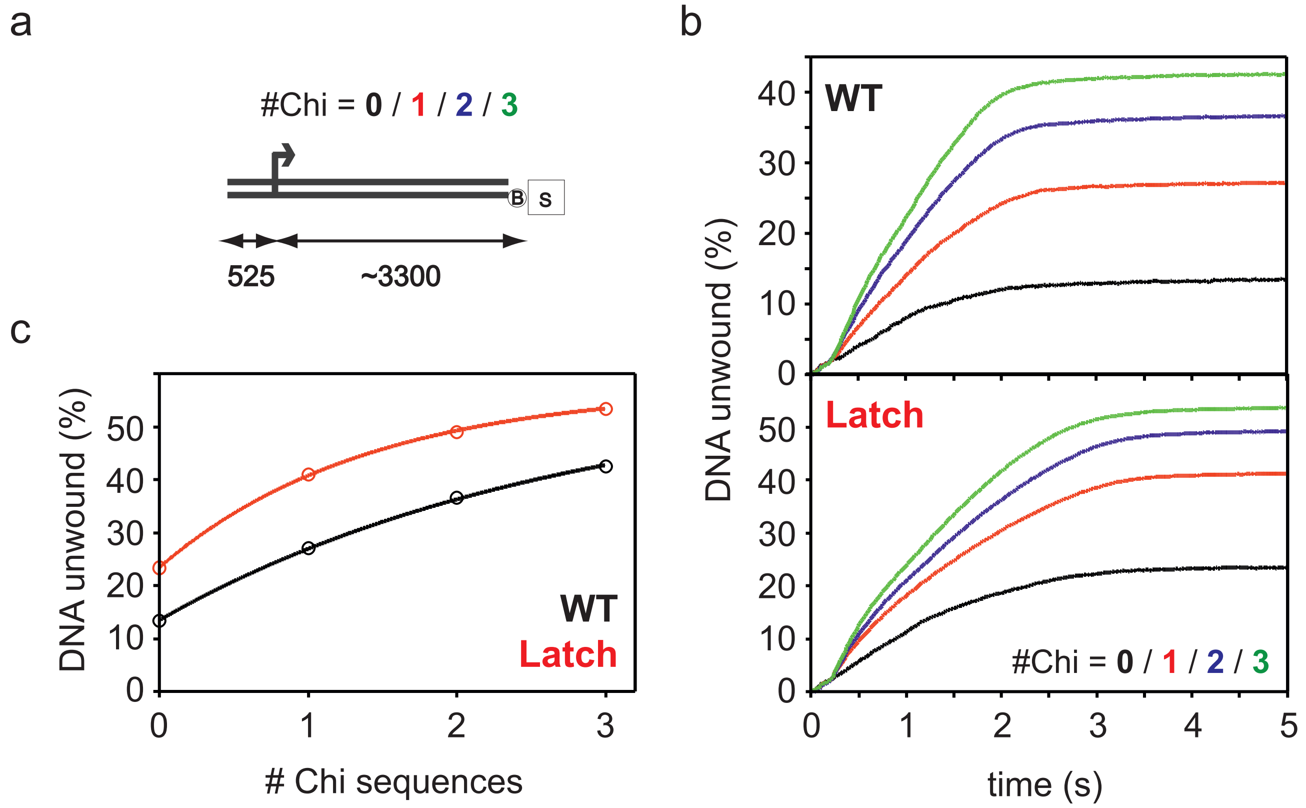

Supplement: SUPPLEMENTARY DATA [file supp_gkv1543_nar-03029-v-2015-File003.pdf]
